# Epitope alteration by small molecules and applications in drug discovery†

**DOI:** 10.1039/d2sc02819k

**Published:** 2022-06-28

**Authors:** Biyue Zhu, Jing Yang, Richard Van, Fan Yang, Yue Yu, Astra Yu, Kathleen Ran, Keyi Yin, Yingxia Liang, Xunuo Shen, Wei Yin, Se Hoon Choi, Ying Lu, Changning Wang, Yihan Shao, Liang Shi, Rudolph E. Tanzi, Can Zhang, Yan Cheng, Zhirong Zhang, Chongzhao Ran

**Affiliations:** Athinoula A. Martinos Center for Biomedical Imaging, Department of Radiology, Massachusetts General Hospital/Harvard Medical School Charlestown Boston Massachusetts USA 02129 cran@nmr.mgh.harvard.edu +1617-643-4886; Key Laboratory of Drug Targeting and Drug Delivery Systems, West China School of Pharmacy, Sichuan University Chengdu 610041 China yancheng@scu.edu.cn +86-28-85501566; Department of Chemistry and Biochemistry, University of Oklahoma Norman OK 73019 USA; Department of Chemistry and Chemical Biology, University of California Merced California 95343 USA; Genetics and Aging Research Unit, McCance Center for Brain Health, MassGeneral Institute for Neurodegenerative Disease, Department of Neurology, Massachusetts General Hospital/Harvard Medical School Charlestown Boston Massachusetts USA 02129 Zhang.Can@mgh.harvard.edu +1617-724-9850; Department of Systems Biology, Harvard Medical School Boston Massachusetts USA 02115

## Abstract

Small molecules and antibodies are normally considered separately in drug discovery, except in the case of covalent conjugates. We unexpectedly discovered several small molecules that could inhibit or enhance antibody–epitope interactions which opens new possibilities in drug discovery and therapeutic modulation of auto-antibodies. We first discovered a small molecule, CRANAD-17, that enhanced the binding of an antibody to amyloid beta (Aβ), one of the major hallmarks of Alzheimer's disease, by stable triplex formation. Next, we found several small molecules that altered antibody–epitope interactions of tau and PD-L1 proteins, demonstrating the generality of this phenomenon. We report a new screening technology for ligand discovery, screening platform based on epitope alteration for drug discovery (SPEED), which is label-free for both the antibody and small molecule. SPEED, applied to an Aβ antibody, led to the discovery of a small molecule, GNF5837, that inhibits Aβ aggregation and another, obatoclax, that binds Aβ plaques and can serve as a fluorescent reporter in brain slices of AD mice. We also found a small molecule that altered the binding between Aβ and auto-antibodies from AD patient serum. SPEED reveals the sensitivity of antibody–epitope interactions to perturbation by small molecules and will have multiple applications in biotechnology and drug discovery.

## Abstract 2

A screening platform based on epitope alteration for drug discovery (SPEED).

## Graphical abstract

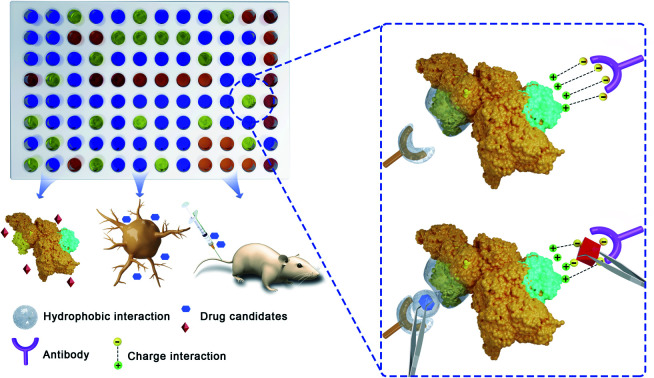

## Introduction

Antibody–antigen interactions often have binding affinities in the low nM range and been extensively applied in basic research, biomarker-based diagnosis and therapeutics. This high affinity is due in part to a large buried surface area with exquisite complementarity. As a result, the antibody–antigen bond can resist large changes in salt, pH *etc.* Here, we consider only protein antigens, whose binding surface is termed an epitope.^1^ Small molecules bind to proteins with much smaller buried surface areas and as a result, are expected to lack the ability to interfere in antibody–epitope binding due to a limited surface area for interaction and a relatively low affinity to an epitope in the absence of other molecules.^2^ Consequently, identifying small molecules to modulate the antibody–epitope interaction has been largely overlooked and its potential applications have been rarely explored.

Over the past decade, our research group has been working on the development of imaging probes and therapeutics for amyloid beta (Aβ), one of the major hypothesized pathogenic targets in Alzheimer's disease (AD).^3–5^ In the course of our studies, we accidentally discovered several ligands that could inhibit or enhance antibody recognition of Aβ epitopes, suggesting the potential of small molecules to alter the binding between the epitope and antibody. Small molecule binding to Aβ has precedents, for example positron emission tomography (PET) tracers for Aβ exhibit binding affinities in the nM region, similar to antigen–antibody interactions.^6,7^ Previous data indicate that an epitope with 15–25 residues has an area of 600–1000 Å^2^.^8^ The minimal region of an epitope for antibody recognition is about 600 Å^2^, which is equivalent to less than 500 Daltons.^9,10^ Recently, Quevedo *et al.* identified small molecule inhibitors for RAS protein by a competitive SPR assay with anti-mutant RAS antibody fragment.^10^ In addition, allosteric small molecules could exhibit an epitope alteration effect due to conformational changes.^2^ Based on our discovery and previous reports, we speculated that the alteration of antibody–epitope recognition could be used to identify small molecule hits for a target-of-interest.

Charges and hydrophobicity are the major interacting forces for epitope–antibody or epitope–ligand interaction.^11^ In the present study, we investigated antibody binding before and after altering the charges and hydrophobicity of an epitope with small molecule ligands. Aβ was selected as the major model protein for several reasons. (1) AD is a currently incurable neurodegenerative disorder and Aβ is one of the primarily hypothesized pathogenic targets and diagnostic biomarker in AD. Using ligands to target Aβ and modulate Aβ-related biofunctions has attracted extensive attention for seeking potential AD therapy.^12^ (2) Charge and hydrophobicity are major determinants driving Aβ misfolding and are closely associated with its toxicity, enzyme activation, oxidative stress, neuroinflammation and production of auto-antibodies.^13–17^ (3) Antibodies for various known Aβ epitopes are already commercially available.^18^ In addition, PD-L1 protein and tau protein were used as additional examples to further demonstrate that the epitope alteration is likely a common but less-explored biological phenomenon.

From our studies, we discovered that small molecules could inhibit or enhance antibody–antigen binding. Based on this discovery, we proposed a screening platform based on epitope alteration for drug discovery (SPEED). As a proof-of-concept study, the SPEED strategy was validated through screening of a library of 1047 compounds to seek lead compounds for Aβ binding. From the results of the SPEED screening, we selected two hits (GNF 5837 and obatoclax) for further validation. We confirmed that GNF 5837, a TrkA inhibitor, could effectively reduce Aβ aggregation. We also demonstrated that obatoclax, an inhibitor of Bcl-2 protein,^19^ could be re-positioned as a fluorescence imaging agent for preliminary screening of AD *via* near infrared fluorescence ocular imaging (NIRFOI). In addition, we demonstrated that a small molecule could alter the binding between Aβ and auto-antibodies from AD patient serum.

## Results

### Small molecule inhibitors for epitope–antibody interaction

**Fig. 1:**
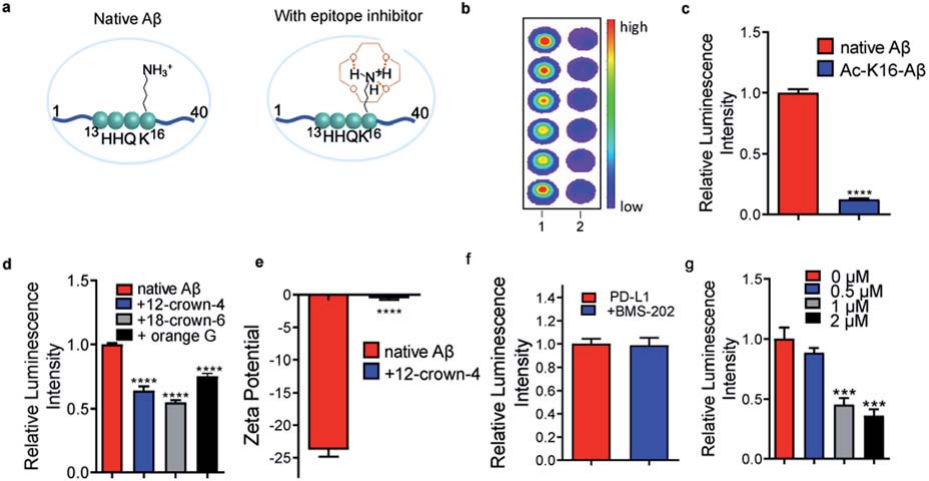
Small molecule inhibitors. (a) Illustration of an epitope before and after charge/electrostatic modulation with 12-crown-4. (b) and (c) Dot blot (b) and quantitative analysis (c) for native Aβs and acetylated-K16 Aβs (Ac-K16-Aβ) using 6E10 antibody (*n* = 6). Ac-K16-Aβs showed decreased signals compared with non-modified Aβs, indicating that the positive charge on K16 is critical for 6E10 antibody recognition. (d) Dot blot results for native Aβs with or without 12-crown-4, 18-crown-6 and orange G using 6E10 antibody (*n* = 6). The addition of 12-crown-4 showed decreased signals of native Aβs compared to the non-treated group, suggesting that 12-crown-4 was capable of altering the charge status of the epitope. 18-crown-6 and orange G showed similar inhibition effects on binding to 12-crown-4. (e) Zeta potential of Aβs with or without 12-crown-4. The zeta potential of Aβs significantly increased after interacting with 12-crown-4, further confirming the charge alteration of the epitope. (f) and (g) Quantitative analysis of dot blot assay for PD-L1 protein with BMS-202 (f) and different concentrations of WL12 (g) using anti-PD-L1 antibody [28-8], whose epitope lies within 19–239 a.a. of PD-L1 (*n* = 6). The readout from WL12 treatment significantly decreased while BMS-202 displayed no obvious change compared to the control.

Charge/electrostatic interactions on an epitope play critical roles in antibody recognition by cation–π interaction, anion–π interaction and hydrogen bond formation.^11^ Previously we found that 12-crown-4 could attenuate the aggregation of Aβs.^20^ Reportedly, crown ethers could form hydrogen bonds with positively charged amino groups (*e.g.* lysine, arginine, histidine) to “shield” the charge,^21^ and the sequence of Aβ peptides contains an important lysine (K16), a crucial amino acid that forms inter-sheet salt bridges and promotes misfolding of Aβ.^14,22^ Thus, we hypothesized that 12-crown-4 could interact with the positively charged ε-amino of K16 *via* forming hydrogen bonds and this interaction could alter the surface charge of Aβ, which could consequently inhibit the binding between the epitope and the corresponding antibody (Fig. 1a).

First, to validate whether an alteration of the surface charge of the Aβ peptide can modulate antibody binding, ε-amino of K16 was acetylated (Ac-K16-Aβ) to turn positively charged K16 into its neutral form, and the chemiluminescence readouts from dot blotting with native Aβ peptide and Ac-K16-Aβ were compared using 6E10 antibody, which is reactive to 1–16 amino acids (a.a.). Indeed, much lower intensity was observed from the Ac-K16-Aβ group than that from the non-modified Aβ group (Fig. 1b and c). In addition, we mutated lysine K16 to glycine on Aβ (K16G-Aβ) and performed the same assay. The mutation causes similar signal decrease (ESI Fig. 1a†). These data indicate that the positive charge on K16 is critical for the binding between Aβ and 6E10 antibody. Next, 12-crown-4 was used to investigate whether it could have similar effects on the binding to the acetylation of K16. After mixing 12-crown-4 with peptides (native Aβ, Ac-K16-Aβ), the intensity of native Aβ was significantly decreased, while no obvious signal change of Ac-K16 Aβ was observed (Fig. 1d and ESI Fig. 1b†), suggesting that 12-crown-4 was capable of altering the charge status of the epitope. In addition, we used its analogues icotinib and 18-crown-6 for the same test. 18-crown-6 showed a similar inhibition effect on binding to 12-crown-4, while icotinib showed no significant inhibition effect (Fig. 1d and ESI Fig. 1c†). The ineffectiveness of icotinib is probably due to its relatively bulky structure to hinder the interaction with the ε-amino of K16 of Aβs. Furthermore, orange G was used as an additional example for altering K16, as previous X-ray analysis of the atomic structure indicated that the negatively charged sulfonic acid groups of orange G could interact with positively charged K16.^14^ As expected, orange G showed similar results to crown ethers (Fig. 1d). To exclude the potential interaction of small molecules with the antibodies, we performed chemiluminescence assay and fluorescence polarization assay to test the signal of HRP or Alexafluo488 labelled primary or secondary antibody before and after compound addition. No significant readout change was found, indicating that the signal changes resulted from Aβ epitope alteration (ESI Fig. 1d–g†). The altering of the surface charge of Aβ by 12-crown-4 was further validated by zeta potential assays, which have been regularly used to measure changes of surface charge status.^23^ As expected, the zeta potential of Aβ significantly increased after interacting with 12-crown-4, further confirming the charge/electrostatic alteration within the epitopes (Fig. 1e). Collectively, our results indicated that the positive charge on K16 was essential for the antibody binding and it could be inhibited by small molecules such as crown ethers and orange G.

To validate whether epitope alteration can be observed beyond Aβs, programmed death-ligand 1 (PD-L1) was selected as another model protein to identify potential epitope inhibitors. PD-L1 is a membrane-bound protein expressed on the surface of tumor cells that inhibit T cell activation by binding to one of its complementary ligands PD-1.^24^ Recently, blocking the PD-1/PD-L1 pathway has become one of the most promising strategies in cancer therapy and several monoclonal antibodies are currently in the market or in the process of FDA approval.^25,26^ Anti-PD-L1 antibody [28-8], which targets the specific extracellular domain of Phe19-Thr239 of PD-L1, and two classes of reported PD-L1 inhibitors including small molecule BMS-202 and cyclo-peptide WL12 were selected for the testing.^25,26^ As shown in Fig. 1f and g, mixing BMS-202 with PD-L1 protein displayed no obvious change compared to the blank control in the dot blot assays. This may arise from the flat and large interface of this epitope that makes it even more difficult for BMS-202 to compete with the binding.^2^ In contrast, the readout of the WL12-treated group showed a significantly decreased signal, indicating the inhibiting role of WL12 for the Phe19–Thr239 epitope. Fluorescence polarization assay showed no significant readout change of WL12 with an AlexaFluo488 labelled primary or secondary antibody (ESI Fig. 2a and b†). The failure of BMS-202 and the effectiveness of WL12 to interact with 19–239 a.a. epitope indicates that a suitable epitope region for interaction is the premise of epitope inhibition with small molecules. Furthermore, we used ANS (1-anilino-8-naphthalenesulfonate) fluorescence assay, which is commonly used for measuring protein surface hydrophobicity, to investigate the hydrophobicity change.^27^ By plotting normalized fluorescence intensity against ANS concentrations, we found that the interaction of WL12 to PD-L1 caused change of epitope hydrophobicity (ESI Fig. 2c†).

### Small molecule binding enhancers for Aβ_17–24_

**Fig. 2:**
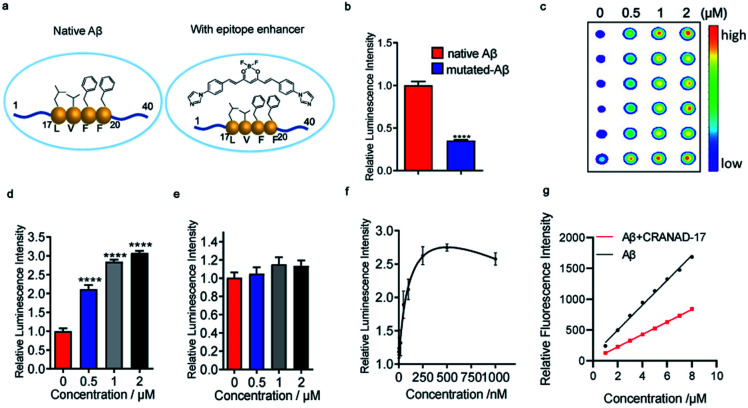
CRANAD-17 as a small molecule enhancer for Aβ_17–24_. (a) Illustration of an epitope before and after hydrophobicity modulation with CRANAD-17. (b) Quantitative analysis for native Aβs and mutated Aβs (F19G, F20G-Aβ) by using 4G8 antibody (*n* = 6). Mutated Aβs showed decreased signals compared with non-modified Aβs, indicating that hydrophobicity interaction is crucial for 4G8 antibody recognition. (c–e) Dot blot assay and quantitative analysis for Aβs with different concentrations of CRANAD-17 by using 4G8 (c and d) and 6E10 (e) antibodies (*n* = 6). CRANAD-17 displayed significantly enhanced chemiluminescence signals from 4G8 antibody, which is more robust than that from 6E10 antibody, indicating that the enhancing effect of CRANAD-17 is specific on epitope Aβ_17–24_. (f) Concentration dependent profile of CRANAD-17 (1–1000 nM) with Aβs (1 µM) determined by the dot blot assay. Half maximal effective concentration (EC_50_ = 121.0 nM) was calculated by plotting the relative luminescence intensity of each group (*n* = 6) using GraphPad Prism 8.0 with nonlinear one-site binding regression. (g) Normalized fluorescence intensity of the hydrophobicity probe ANS (0–10 µM) after adding 5 µM Aβs with or without 10 µM CRANAD-17. The curve showed a significantly changed slope (*P* < 0.0001), indicating that surface hydrophobicity changes upon mixing with CRANAD-17.

Previously we reported that curcumin analogue CRANAD-17 could bind to Aβ species and attenuate the cross-linking of Aβs. ^1^H NMR spectra showed that CRANAD-17 could interact with the Aβ_16–20_ fragment, which covers the core hydrophobic region of Aβ peptides.^28^ The curcumin scaffold of CRANAD-17 serves as the anchor moiety and the benzene and imidazole rings on both sides provide hydrophobic interaction with the core fragment of Aβ. We hypothesized that CRANAD-17 binding could alter the hydrophobic properties around Aβ_17–24_, which can be recognized by the 4G8 antibody (Fig. 2a). First, we used glycines to replace the phenylalanines (F19 and F20), the highly hydrophobic amino acid on the Aβ, and the chemiluminescence readouts from dot blotting with native Aβ peptide and mutated Aβ (F19G, F20G) were compared using 4G8 antibody, which is reactive to 17–24 amino acids (a.a.). As expected, the mutated Aβ group showed significantly decreased intensity compared with the native Aβ group (Fig. 2b), indicating the critical role of phenylalanines in the binding between Aβ and 4G8 antibody. Next, the chemiluminescence readouts from Aβs with or without CRANAD-17 were compared using 4G8 antibody. Thioflavin T (ThT), a standard dye for Aβ plaques but not specific to Aβ_17–24_,^29^ was used as a negative control. With the addition of CRANAD-17, surprisingly, all Aβ species displayed significantly enhanced chemiluminescence intensities detected by 4G8 antibody (Fig. 2c and d and ESI Fig. 3a–d† for oligomers and aggregates). In addition, no significant binding was found when incubating these compounds with primary or secondary antibody only (ESI Fig. 3e–h†). Although we expected CRANAD-17 to change the readouts, the increase in chemiluminescence intensity was considerably surprising. In contrast, ThT showed no apparent changes in the readout for Aβ species, which is in accordance with a previous study revealing that ThT primarily binds in channels running parallel to the long axis of Aβ fibrils (ESI Fig. 4a and b†).^29^ To investigate the specificity and sensitivity of this method, 6E10 antibody (reactive to Aβ_1–16_) was used for comparison. As shown in Fig. 2d and e, CRANAD-17 displayed more robust increase in signal from the 4G8 antibody than that from the 6E10 antibody, confirming that the alteration effect of CRANAD-17 is specific on epitope Aβ_17–24_. Furthermore, a concentration-dependent titration was performed by using 4G8 antibody for the detection. As shown in Fig. 2f and ESI Fig. 5,† the enhancement was in a concentration-dependent manner, and the EC_50_ was 121.0 nM.

To investigate whether CRANAD-17 could lead to changes of epitope hydrophobicity, fragment Aβ_17–24_ and the full-length of Aβ were used in the ANS fluorescence assay.^27^ As shown in Fig. 2g and ESI Fig. 6a,† the fluorescence profile of Aβ significantly changed upon mixing with CRANAD-17 with a 22 nm redshift in the emission spectra. The decreased fluorescence intensity indicates lower hydrophobicity,^27^ which causes a redshift of the wavelength due to dipole–dipole interaction.^30^ Interestingly, the hydrophobicity of Aβ_17–24_ also decreased in the presence of CRANAD-17 (ESI Fig. 6b†). Taken together, CRANAD-17 could alter the hydrophobicity of Aβ_17–24_ and ultimately alter the hydrophobicity of Aβ_1–40_ and thus change the binding behaviour of the antibody. However, it is still not clear how the changes of the binding environment alter the antibody binding. It is likely that the match of the hydrophobicity between the antibody and epitope plays a crucial role in their binding behavior. In other words, either decreased or increased epitope hydrophobicity could adjust to the best range for antibody binding and lead to an enhanced signal.

The above results of CRANAD-17 were obtained by using purified proteins. However, it was not clear whether CRANAD-17 has a similar modulation effect on Aβs in a biologically relevant environment. In this regard, the medium from 7PA2 cells, which is a familiar APP mutation transfected cell line that secretes Aβ_1–40_ and Aβ_1–42_, was used.^31^ Despite the low concentration of Aβ in cell media, the epitope alteration effect could still be detected (ESI Fig. 7a and b†). In addition, transgenic mice brain homogenate displayed enhanced readout after interacting with CRANAD-17 (ESI Fig. 7c and d†). These data suggest that CRANAD-17 could execute its modulation effect in a biologically relevant environment.

Over the past several years, we have synthesized numerous curcumin analogues CRANAD-*X*s.^32^ To investigate whether these analogues have a similar effect to CRANAD-17, we performed testing with CRANAD-*X*s (*X* = −3, −25, −44 and −102). As shown in ESI Fig. 8,† these compounds showed enhanced 4G8 antibody recognition to Aβs. Interestingly, CRANAD-44, 102 could not change the 4G8-epitope interaction for insoluble Aβ aggregates (ESI Fig. 8e and f†). This result is consistent with our previous report, in which we demonstrated that CRANAD-102 had good selectivity for soluble Aβs over insoluble Aβ aggregates.^32^

### Mechanism studies for epitope binding enhancers

**Fig. 3:**
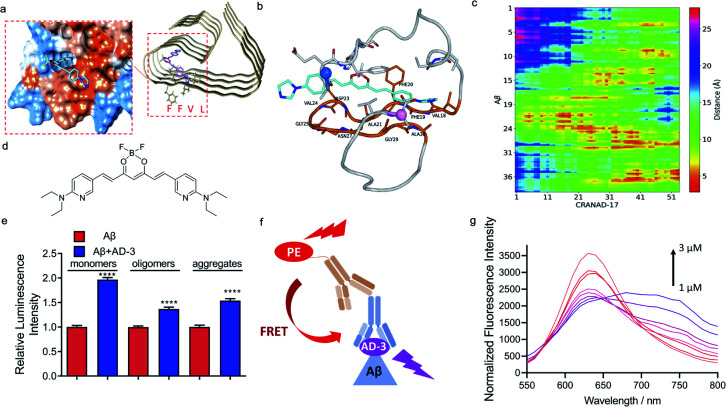
Mechanism studies for epitope enhancers. (a) Molecule docking of the binding poses of CRANAD-17 with Aβs (PDB: 5OQV). The protein surface is colored according to amino acid hydrophobicity (red: hydrophobic, blue: hydrophilic). The results suggest that CRANAD-17 binds to the hydrophobic moiety LVFF *via* the interaction of benzene and imidazole rings within the hydrophobic pocket of Aβs. (b) A representative MD snapshot of CRANAD-17 binding with Aβ monomers. CRANAD-17 is highlighted in cyan, and all residues on Aβ that are within 5 Å of CRANAD-17 are shown in the stick representation. The residue loop 17–30 is highlighted in orange, and those that are within 5 Å of CRANAD-17 are also labelled. CA atoms on N and C termini are highlighted in marine and pink, respectively. (c) The distance map between each atom of CRANAD-17 and all heavy atoms in Aβ, *y*-axis is labelled as the peptide residue index. (d) The structure of CRANAD-3. (e) Quantitative data of dot blot assays for 1 µM Aβ_1–40_ monomers, oligomers and aggregates with or without 2 µM of CRANAD-3 by using 4G8 antibody. CRANAD-3 could change the 4G8 antibody recognition and lead to increased readout for all Aβ species. (f) Illustration of the FRET assay. If CRANAD-3 could bind with the epitope of 4G8 antibody on Aβ monomers and form a ternary complex, the PE-labelled secondary antibody could serve as the donor molecule and excite CRANAD-3. (g) Fluorescence spectra of the mixture of Aβ monomers, 4G8 antibody and PE-labelled secondary antibody titrated with CRANAD-3 (1–3 µM). The typical FRET phenomenon is shown wherein the emission peaks of PE decreased while the emission peaks of CRANAD-3 simultaneously increased.

Given that the phenomenon of increased antibody recognition in the presence of CRANAD-17 is unexpected, we set out to investigate the interaction mechanism. To this end, computational studies with CRANAD-17 were performed based on Aβ fibril structure models 5OQV and 2LMO from the Protein Data Bank (PDB),^33^ and ThT was used for comparison. Consistent with our previous NMR studies, both molecular docking data suggested that CRANAD-17 binds to the hydrophobic moiety LVFF *via* the interaction of benzene and imidazole rings within the hydrophobic pocket of Aβ (Fig. 3a). In contrast, docking results of ThT showed dominant sites around the grooves formed by ^10^YEVHHQK^16^ in the 5OQV model and around the C-terminal in the 2LMO model (ESI Fig. 9 and Table 1†).

Next, molecular dynamics (MD) simulations of Aβ monomer bound to CRANAD-17 (denoted as Aβ_1–40_-CRANAD17) in water were performed. Five independent simulations with different initial configurations and velocities were conducted using the GROningen MAchine for Chemical Simulations (GROMACS), version 2018.4, package to improve statistical efficiency.^34^ As shown in Fig. 3b, CRANAD-17 interacted closely with Aβ around residues 17–30. Among all five simulations, four of them showed that CRANAD-17 bound to the peptide near the region of residues 17–30. Given the hydrophobic nature of CRANAD-17, especially around the benzene rings at the two ends, it is not surprising to see that it tends to favorably interact with the loop of residues 17–30 where residues are dominantly hydrophobic. We then computed the distances between CRANAD-17 and all heavy atoms in Aβ (Fig. 3c), and confirmed that CRANAD-17 tends to have the closest distance (shown as red regions in the distance map in Fig. 3b) to the residue loop 17–30. Interestingly, the two termini from Aβ (N-terminus and C-terminus) are also in close proximity to CRANAD-17, and it can be explained by the fact that CRANAD-17 is an extended stiff molecule with identical aromatic hydrophobic moieties at both ends, which can be approached by the flexible termini of Aβ. At the N terminus residue Phe4 interacts strongly with CRANAD-17 *via* π–π interaction, while the residues Val39 and Val40 at the C terminus stay close to CRANAD-17 due to hydrophobic interactions. These results suggested that CRANAD-17 may form a ternary complex with 4G8 antibody and Aβs, and thus stabilize the binding.

To further explore the enhanced antibody–epitope binding, we used the Förster resonance energy transfer (FRET) technique to validate the formation of the ternary complex of antibody–small molecule–epitope. FRET is a widely utilized technology for studying protein–protein interaction.^35^ When the distance between the donor fluorophore and the acceptor fluorophore is in proximity (1–10 nm) due to the binding of the two proteins, FRET occurs. First, we labelled a secondary antibody with Alexa fluo 488 to serve as a donor moiety and CRANAD-17 as the acceptor moiety. We titrated the mixture of Aβ monomers, 4G8 antibody and the corresponding secondary antibody with different concentrations of CRANAD-17. Although a FRET phenomenon can be observed (ESI Fig. 10†), the FRET efficiency is low due to the weak fluorescence of CRANAD-17. In this regard, we used CRANAD-3, which is a close analogue of CRANAD-17 (Fig. 3d), and has much stronger fluorescence than CRANAD-17, as a fluorescence probe for Aβ species,^4^ to monitor the antibody–epitope–CRANAD-3 interaction. Previous NMR results showed the binding of CRANAD-3 to the ^17^LVFF^20^ fragment,^4^ suggesting that CRANAD-3 may possess similar epitope alteration properties to CRANAD-17. First, dot blot assay was conducted to confirm the epitope enhancer role of CRANAD-3. As we expected, CRANAD-3 showed a significant enhancing effect upon interacting with Aβ species (Fig. 3e). To investigate whether CRANAD-3 could form a ternary complex with 4G8 antibody and Aβs, we performed FRET studies with the solution mixture of CRANAD-3, 4G8 antibody and Aβ_1–40_ monomers. With suitable fluorescence properties, PE labelled secondary antibody was selected as the donor fluorophore and CRANAD-3 was selected as the acceptor fluorophore (Fig. 3f). Different concentrations of CRANAD-3 were used to titrate the mixture of Aβ monomers with 4G8 antibody and the corresponding secondary antibody. The mixture without Aβ monomers was used as the control and the final spectra were recorded after normalizing the background. With increased concentrations of CRANAD-3, the emission peaks of PE apparently decreased while the emission peaks of CRANAD-3 with Aβ simultaneously increased (Fig. 3g). This typical FRET phenomenon further validated that the enhanced antibody recognition was due to the formation of the ternary complex of CRANAD-3, Aβs and 4G8 antibody.

### Identification of epitope enhancers for tau

Inspired by the above results, we set out to identify small molecular modulators for tau protein, which is another hallmark in AD and share the similar β-sheet structure with Aβs. Tau protein is a microtubule-associated protein with a critical role in several tauopathies including AD, Parkinson's disease and Pick's disease.^36^ Targeting tau protein with small molecules or antibodies has been of increasing interest in manipulating tauopathies.^37^ The VQIVYK segment of tau protein has been shown to drive tau aggregation and thus is considered an important target for seeking inhibitors for inhibiting tau fibrilization.^38^ 8E6/C11 antibody, whose epitope lies within 209–224 a.a. of human tau (3-repeat isoform RD3) containing the VQIVYK segment, can provide a suitable epitope region for small molecule interaction and thus was selected as the antibody for identifying potential epitope modulators. Several commercially available tau inhibitors, tracers and dyes including ATPZ, T-807 and thioflavin S (ThS) were selected to conduct the assay.^39–41^ As shown in ESI Fig. 11,† ThS showed significantly increased signal upon interacting with tau protein, which is in accordance with previous studies that indicate ThS could bind to the VQIVYK-containing fragment.^41^ In contrast, the modulation effect of ATPZ and T-807 on the VQIVYK-containing epitope is negligible, indicating that these compounds cannot affect this epitope.

### Screening new compound leads for Aβs with SPEED

**Fig. 4:**
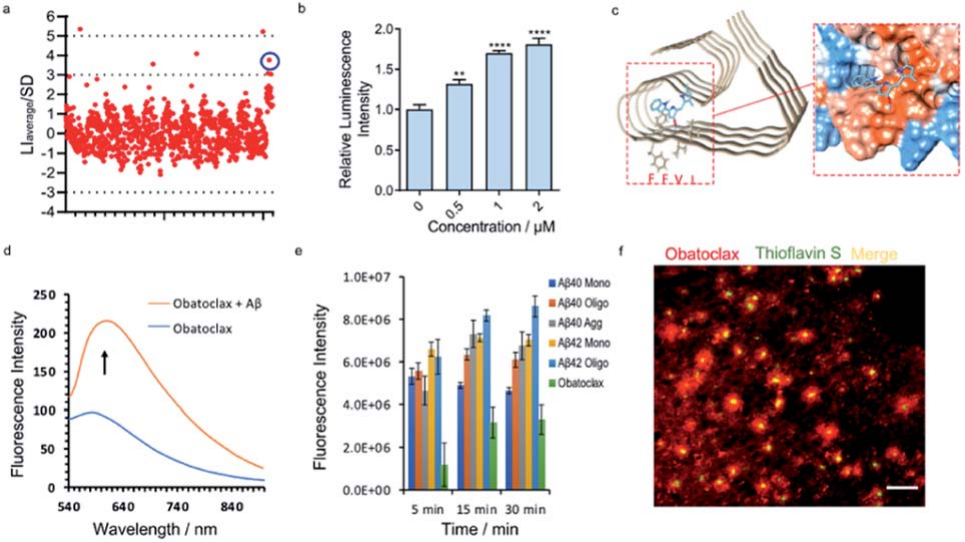
SPEED screening for seeking obatoclax as a new lead for Aβs. (a) Representative dot blot screening results with a library of 1047 compounds. Obatoclax (in blue circle) showed obviously increased signal for Aβs with 4G8 antibody. (b) Quantitative analysis of the dot blot assay of Aβs with different concentrations of obatoclax by using 4G8 antibody (*n* = 6). The results showed moderately increased signal for Aβs upon mixing with obatoclax, confirming the alteration of the epitope. (c) Molecular docking of the binding poses of obatoclax with Aβs (PDB 5OQV). The protein surface is colored according to the amino acid hydrophobicity (red: hydrophobic, blue: hydrophilic). The interacting sites of obatoclax contain the ^17^LVFFAEDV^24^ segment. (d) Fluorescence emission spectra of obatoclax with or without Aβ aggregates (*E*_x_/*E*_m_ = 570/620 nm). (e) Quantitative analysis of the fluorescence intensity of obatoclax with different Aβ species. (f) *In vitro* fluorescence staining of obatoclax on transgenic mice brain slices. The substantial staining of plaques was confirmed by co-staining with a standard dye such as thioflavin S.

Based on the encouraging results above, we hypothesized that the epitope alteration phenomenon can be used to discover new leads for drug candidates and seeking imaging ligands. We proposed a screening platform based on epitope alteration for drug discovery (SPEED), which provides label-free and site-specific screening for various functional purposes. As a proof-of-concept study, we used Aβ_1–40_ monomers as the target. The structure of Aβ can be classified into a hydrophilic N-terminal region (10–16 a.a.), central hydrophobic β1 region (17–21 a.a.), hydrophilic turn region (22–28 a.a), and hydrophobic β2 region (29–40/42 a.a.).^17,42^ We selected Aβ_17–24_ as the targeted epitope as targeting the main core of the Aβ containing KLVFF segment has been utilized as the mechanism for developing anti-aggregation inhibitors and also fluorescence probes based on the restriction of the rotation effect.^43,44^ We performed the SPEED screening with a library of 1047 compounds and antibody 4G8 was used for targeting Aβ_17-24_. The compounds with an absolute value of average chemiluminescence intensity higher than 3xSD (standard derivation) were selected as hits (Fig. 4a, and ESI Fig. 12†). The screening results showed 7 positive results and further screening confirmed two robust hits of GNF-5837,^45^ a tropomyosin receptor kinase (Trk) inhibitor, and obatoclax, an inhibitor of Bcl-2 protein.^19^

We first validated the binding of GNF-5837 to Aβs. GNF-5837 showed excellent dose-dependence epitope binding, evidenced by the dot blot assay with 4G8 antibody (ESI Fig. 13a†). Interestingly, with increased concentrations of GNF-5837, the signal increased and then drastically decreased when 6E10 antibody was used for detection (ESI Fig. 13b†), this suggests an allosteric role of GNF-5837 that causes conformational change of Aβs. Next, molecular docking studies were performed. The results revealed that GNF-5837 bound with ^17^LVFFAEDVGSNKGAI^31^ (ESI Fig. 13c†). Furthermore, we set out to explore whether GNF-5837 can be used for anti-aggregation purposes. Given that CRANAD-3 and GNF-5837 share the same binding site containing Aβ_17–24_, we speculated that the two compounds could compete with each other. Indeed, we obtained a classical competitive binding curve of fluorescence intensity of CRANAD-3 with Aβ monomers (*K*_i_ = 4.16 ± 1.04 nM, ESI Fig. 13d†), further confirming the reliability of our SPEED screening results. In addition, we used ThT to monitor Aβ aggregation behaviors with or without GNF-5837. As expected, the addition of GNF-5837 significantly reduced Aβ aggregation, as indicated by the decreased fluorescence intensity of ThT (ESI Fig. 13e†). Interestingly, in the course of our experiments, we observed that the binding of GNF-5837 with Aβ_1–40_ monomers could prevent the aggregation of GNF-5837 itself during the incubation period (ESI Fig. 13f†), evident through the precipitate from the solution of GNF-5837 compared with the clear solution of the Aβ/GNF-5837 mixture. Moreover, the fluorescence spectra of CRANAD-3 with Aβ monomers, oligomers and aggregates with or without GNF-5837 were recorded (ESI Fig. 14†). The competitive binding phenomenon further confirmed the strong binding of GNF-5837 to all species of Aβ.

To validate the binding of obatoclax to Aβs, a concentration-dependent titration was performed. As expected, obatoclax showed excellent dose-dependence for the binding between 4G8 and Aβ monomers, and could strengthen the binding of 4G8 antibody to Aβ oligomers and aggregates (Fig. 4b, ESI Fig. 15a and b†). Interestingly, obatoclax also showed a moderate epitope alteration effect detected by 6E10 antibody but not as strong as the 4G8 group (ESI Fig. 15c†). To further validate that obatoclax is able to bind to the epitope of Aβ_17–24_, molecular docking studies were performed. The results confirmed that the interacting sites of obatoclax contain Aβ_17–24_ (Fig. 4c, ESI Fig. 16 and Table 1†). We noticed that the structure of obatoclax was partially overlapped with ThT, and thus it could possess fluorescence properties and could be used for imaging purpose. To investigate whether obatoclax could fluorescently detect Aβ species, we performed *in vitro* solution tests. As shown in Fig. 4d, the fluorescence intensity of obatoclax was significantly increased upon mixing with Aβ aggregates (*E*_x_/*E*_m_ = 570/620 nm). We also performed similar tests with Aβ monomers and oligomers, and found that the fluorescence intensity was significantly increased (Fig. 4e). *In vitro* fluorescence staining with obatoclax on transgenic mice brain slices showed highlighted plaques with excellent contrast, and these plaques were consistently labelled with a standard dye such as thioflavin S for colocalization (Fig. 4f).

**Fig. 5:**
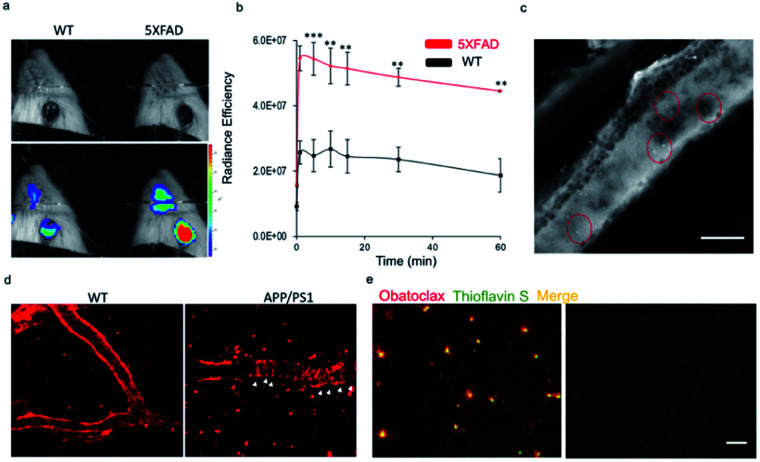
*In vivo* fluorescence imaging applications of obatoclax. (a and b) NIRFOI for 5xFAD mice and WT mice with i.v. injection of obatoclax (4 mg kg^−1^). Representative photographic images of a WT and a 5XFAD mouse (a) and quantitative analysis of the NIRFOI signals at different time points (b). (c) *Ex vivo* retinal microscopic imaging of slices from a 5xFAD mouse. The plaques are indicated by the red circles. Scale bar: 200 µm. (d) Representative images of *in vivo* two-photon imaging of APP/PS1 mice and WT after i.v. injection of obatoclax (4 mg kg^−1^). Plaques and CAAs can be clearly observed. (e) *Ex vivo* histological staining of brain slices from a 9 month-old 5xFAD mouse and wild-type mouse after 2 mg kg^−1^ obatoclax administration. The brain sections of the 5xFAD mouse treated with obatoclax showed substantial staining of Aβs that were confirmed by subsequent staining of 0.1% thioflavin S, while wild-type mouse brain showed no labelling (scale bar = 50 µm).

Recently, we have demonstrated the application of CRANAD-*X* probes in near infrared fluorescence ocular imaging (NIRFOI) for AD mice models, which has great potential for preliminary screening of AD in future clinical studies. With the low molecular weight of 317.4 Daltons and cLog P of 3.14, obatoclax would be favorable for penetrating the blood–brain barrier.^3^ To validate whether obatoclax could be used for NIRFOI, 5xFAD mice (Tg) and age-matched wild-type (WT) mice were i.v. injected with obatoclax (4 mg kg^−1^), and NIRFOI data were captured for 60 minutes. Indeed, the NIRFOI signals were significantly higher in 5xFAD mice than in WT mice (Fig. 5a and b). To further validate the capacity of obatoclax for staining Aβ plaques in eyes, we performed *ex vivo* histological studies, in which a 9 month-old 5xFAD mouse was injected intravenously with obatoclax and sacrificed at 30 minutes after the injection. We dissected the eyes into 20-micron slices, and found that obatoclax could light up the plaques in the retina (Fig. 5c). Our data indicated that obatoclax was capable of penetrating the retinal blood barrier (RBB) and binding to plaques. Taken together, our *in vivo* and *ex vivo* data suggested that obatoclax could be used for NIRFOI, and may have potential for translational studies in the future.

To further validate the imaging capacity of obatoclax *in vivo*, we performed two-photon microscopic imaging with living 20 month-old APP/PS1 and age-matched WT mice, cranial windows of which have been created *via* surgery. An APP/PS1 mouse was i.v. injected with obatoclax (4 mg kg^−1^), and images were captured at 10 minutes after the injections. Apparent plaques and cerebral amyloid angiopathy (CAA) could be clearly observed from the APP/PS1 mice while no such features were found in WT mice, suggesting that obatoclax can bind to Aβs *in vivo* (Fig. 5d). Furthermore, *ex vivo* histological studies were performed, showing excellent staining of Aβ plaques that colocalized with thioflavin S (Fig. 5e).

Collectively, our data suggest that obatoclax could serve as a fluorescence imaging probe for Aβs, and GNF-5837 could potentially be used as an anti-aggregation ligand for Aβs. Taken together, the SPEED could be applied for seeking drug candidates/imaging probes.

### Potential applications of SPEED

To further demonstrate the screening potential of SPEED, a *Z*′ factor test was conducted. A plate with half negative control (no GNF-5837) and half positive control (GNF-5837) was tested by using 4G8 antibody and Aβ_1–40_ monomers as the target. As shown in ESI Fig. 17a and b,† the positive control groups showed a significantly enhanced signal compared with the negative control group. The *Z*′ factor was also calculated, which is the most commonly used parameter to evaluate the quality of an assay before conducting high-throughput screening (*Z*′ factor > 0 are considered valid, *Z*′ factor > 0.5 are considered excellent).^46^ The *Z*′ factor of this assay is 0.36, which may be due to the deviation of the microfiltration plate and limited signal change caused by epitope alteration. Despite the relatively low *Z*′ factor, dot-blotting based screening is a simple and label-free method with inexpensive apparatus. It enables fast immobilization and detection of targets that could be easily implanted into many biomedical laboratories. To further explore the potential of this strategy, we also validated the SPEED concept with advanced immunoassay technologies. Meso Scale Discovery (MSD) assay was first conducted. With GNF-5837 and CRANAD-17 as model drugs and Aβ_1–40_ monomers as the model target, the enhanced antibody recognition could be quantified at the 200 pg mL^−1^ level of sample consumption (ESI Fig. 17c†). We also tested the SPEED concept with fully automated single molecule array (Simoa), a newly established digital ELISA. Simoa is an ultrasensitive method for detecting biomarkers with femtogram per milliliter (fg ml^−1^) level sensitivity. Simoa technology utilizes 50 fL microwells as reaction chambers to isolate and detect single molecule binding on one paramagnetic bead, and the signal is determined digitally with time-lapsed fluorescence intensity quantification (ESI Fig. 17d†).^47,48^ With the Simoa technology, GNF-5837 showed significantly enhanced antibody recognition with only 1 pg mL^−1^ Aβ samples, and CRANAD-17 also showed moderate signal enhancement (ESI Fig. 17e†). Thus, MSD and Simoa could provide strong technical support for further high throughput SPEED screening.

### Epitope alteration with auto-antibody

Aβ has been considered as an antigen that correlates with neuroinflammation and production of anti-Aβ auto-antibody.^16,49^ The serum from healthy individuals and AD patients both contain anti-Aβ auto-antibody.^50^ Currently, a clear mechanism for antigen and auto-antibody is still elusive.^51^ This triggers our interest in studying the effect of our small molecules on auto-antibody. First, we set out to explore whether the small molecule induced epitope alteration effect can be observed with auto-antibody and Aβ. We selected 12-crown-4 and CRANAD-17 to alter the epitopes within Aβ monomers. Specifically, we first incubated Aβ monomers, 12-crown-4 and CRANAD-17, then conducted dot blotting assays using the Aβ auto-antibodies in the human serum from healthy individuals or AD patients as the primary antibody for detection. In this case, no externally added primary antibody was used. We used Aβ/serum (without the compounds) as the control group and as the normalizing baseline for the quantification. As shown in ESI Fig. 18,† the healthy individual serum group showed no significant changes with both compounds. Interestingly, the AD patients' serum group showed significant decrease of normalized signals when incubating with CRANAD-17. These data suggest that healthy individuals and AD patients probably have different Aβ auto-antibodies. Reportedly, Aβ auto-antibody from AD patients recognized the mid-/C-terminal end of Aβ.^16^ This indicated the allosteric effect of CRANAD-17 to the mid-/C-terminal end of Aβ, which is in accordance with our MD results in Fig. 3b that show the exposure of the C-terminal upon Aβ/CRANAD-17 interaction. To further confirm this allosteric interaction, we incubated Aβ monomers and CRANAD-17 in PBS buffer and the mixture was subjected to dot blotting with 12F4 antibody, which is C-terminal specific (targeting Aβ36-42). Indeed, we found that CRANAD-17 could reduce the binding of 12F4 antibody to Aβs (ESI Fig. 19†).

## Discussion

In the present study, we discovered that 12-crown-4, 18-crown-4, and orange G could inhibit 6E10 antibody binding to Aβ aggregates, while CRANAD-*X*s, obatoclax and GNF-5837 could enhance the binding of 4G8 to Aβ species. Moreover, we identified ThS as an epitope enhancer for tau protein and WL12 as an epitope inhibitor for PD-L1 protein, suggesting that epitope alteration is likely a common but less-explored biological phenomenon in antibody–epitope interactions. For a proteinaceous/peptidic antigen, the antibody–antigen interaction could be considered as a type of protein–protein interaction (PPI). When introducing an additional molecule (ligand) that could also bind with the antigen, several possible binding modes exist. (1) Competitive binding mode, which could result in the inhibition of antibody binding. Normally, the dissociation constant (*K*_d_) of an antibody and its antigen is in the range of nanomole (*e.g.* the *K*_d_ of anti-β-Amyloid 17–24 antibody for Aβ_1–40_ monomer is 30.1 nM).^18^ If the ligand, which has a similar range of *K*_d_ (the *K*_d_ of Aβ ligand CRANAD-3 is 24 nM),^4,18^ and antigen are in the competitive mode, a decrease in antibody binding is expected. (2) Allosteric binding mode, which could generate conformational change of the epitope, leading to inhibited (allosteric inhibitors) or enhanced (allosteric activators) antibody binding.^2,52^ (3) Synergic enhancing binding mode, which could result in enhanced antibody binding. Recent findings discovered cooperative roles of ligands (PPI stabilizers or chemical dimerizers) that could bind at the interface and stabilize PPI.^53–55^ Our data showed both inhibition and enhancement for antibody–epitope binding, which indicates different binding effects of these small molecules on the epitopes.

We envisioned that small molecule epitope modulators (including inhibitors and enhancers) could be utilized in a broad scope of research and therapeutic purposes. First, epitope inhibitors or enhancers for monoclonal antibody drugs could provide versatile tools to manipulate their pharmacological effects.^56^ Second, epitope modulators for auto-antibodies have great potential to manipulate immune responses. Epitope enhancers could strengthen antibody–antigen interaction to avoid immune escape of antigens, and epitope inhibitors could block epitopes to inhibit the innate immune attack in autoimmune diseases.^57^ Moreover, epitope modulators could have broad applications in basic research purposes (*e.g.* etiology elucidation, *etc.*). Recently, we also applied SPEED to validate the binding of a donor–acceptor Stenhouse adduct (SHA-2) to hydrophobic binding pockets of Aβs.^58^ Thus, SPEED could be a versatile method for investigating the compound–epitope interaction and for small library screening.

The SPEED strategy has several advantages. It requires no modification/tagging of target proteins and ligands, and various immunoassay technologies are compatible for the SPEED strategy, which could be complementary to current label-free screening methods, including drug affinity responsive target stability (DARTS),^59^ and surface plasmon resonance (SPR) technology.^35^ In addition, we envision that the SPEED could provide a new angle for drug screening. The SPEED strategy takes advantages of prior knowledge of binding pockets or functional sites of protein, which could potentially decrease the laborious screening effort. In the present study, we successfully identified two hits for Aβ. This may be due to our prior knowledge about the hydrophobic pockets of Aβ including Aβ_17–24_ that could provide a preferable binding site for small molecules. Our results suggested that some residues might dominate certain protein functions and thus are potentially important for drug discovery. For example, PPI usually involves a few key residues called “hot spots” that contribute the majority of the binding of protein–protein interface.^2^ In addition, the SPEED would be favorable to screen substitute small molecules for antibody drugs as the macromolecules are defective for intracellular or cerebral targets.^10^

Based on the results from the present study, we proposed the workflow of the SPEED strategy for future studies. (1) Target epitope selection: targeted epitopes are designed based on prior knowledge of functional sites or preferable binding pockets of target proteins. (2) Antibody selection: if the epitope of interest is clear and the corresponding antibody is available, the selection is an easy step. However, if the antibody is not available, it could be produced and purified *via* injecting the antigen into animals to produce antibodies targeting specific epitopes. (3) Drug screening: Simoa, MSD or dot blot assay could be used for screening. With different antibody labelings on beads or plates, MSD and Simoa enable targeted epitope screening or multiplex screening for various epitopes.

The SPEED also has certain limitations, and several obstacles are required to be addressed. For example, prior knowledge of the epitope region within an antigen is needed and the corresponding antibodies should be available. However, as the utilization of antibodies is rapidly developing in both clinical therapeutics and research applications, we believe this limitation could be overcome by advancements in antibody discovery and the epitope mapping process. Furthermore, to select a potential therapeutic site (epitopes), more intensive and comprehensive studies are needed to explore the relationship of a specific epitope and its functions. We believe this limitation could be addressed by the advance of multiplex technology with antibody arrays,^60^ which could provide technical support for screening multiple epitopes. Despite these limitations, the SPEED provides a simple, fast and label-free strategy for identifying ligands that could complement current methods for drug discovery.

Further efforts on the SPEED phenomenon would be highly desirable in characterizing the underlying mechanism, providing rational design criteria and directed SPEED screening for the protein of interest. Further research is currently underway in our group.

## Conclusions

In summary, we discovered several small molecules that could alter the surface properties of amyloid beta (Aβ) epitopes. Consequently, the alteration could lead to inhibition or enhancement of the antibody–epitope recognition. We proved that the enhancement phenomena were caused by the formation of a ternary complex of the Aβ, small molecule, and antibody. In addition, several reported ligands of tau and PD-L1 proteins could also alter the antibody recognition to corresponding epitopes. Based on the above discovery, we proposed a screening platform based on epitope alteration for drug discovery (SPEED). SPEED was successfully applied to seek new ligand leads for Aβ species by discovering GNF-5837 as an Aβ aggregation inhibitor and obatoclax as an Aβ fluorescence probe. We also found a small molecule that altered the binding between Aβ and auto-antibodies from AD patient serum. Our findings disclosed the largely overlooked phenomenon of epitope alteration with small molecules and we demonstrated its application to complement current label-free drug screening strategies.

## Methods

### General information

All reagents were commercial products and used without further purification. CRANAD-*X* compounds were synthesized according to our previously reported procedures, and compounds with purities more than 95% determined by analytical HPLC were used for further evaluation.^28,32^ Aβ_1–40/42_ peptide was purchased from rPeptide, and different forms of Aβ_1–40/42_ including the monomer, oligomer and aggregate were prepared by our previously reported methods.^28^ Acetylated-K16 Aβ_1–40_ and mutated Aβ_1–40_ (K16G-Aβ, F19G, F20G-Aβ) were purchased from GenScript. Purified anti-β-Amyloid 1-16 (clone 6E10), 17–24 (clone 4G8) antibodies were purchased from BioLegend. Tau441 and tau352 were purchased from rPeptide. Anti-Tau (3-repeat isoform RD3) antibody (clone 8E6/C11) was purchased from MilliporeSigma. Recombinant anti-PD-L1 antibody [28-8] and recombinant human PD-L1 protein were purchased from Abcam. HRP-conjugated goat anti-mouse IgG (H + L) secondary antibody was purchased from Invitrogen. 5xFAD and wild type mice were from Rudolph Tanzi Laboratory. All animal experiments were approved by the Institutional Animal Use and Care Committee (IACUC) at Massachusetts General Hospital, and carried out in accordance with the approved guidelines. Healthy human serum and AD patient serum were commercially available samples purchased from Innovative Research. The patients were clinically identified by Innovative Research and detailed information is documented in the ESI, Table 2.†

### Cell culture

7PA2 cells were cultured in Dulbecco's Modified Eagle's Medium/F-12 medium with high glucose (Gibco, USA) supplemented with 2 mM l-glutamine, 200 µg mL^−1^ G418, 100 IU mL^−1^ penicillin, and 100 µg mL^−1^ streptomycin at 37 °C in an atmosphere containing 5% CO_2_.

### Dot blot assay with purified proteins

Nitrocellulose membrane (0.2 µm, Bio-Rad) was soaked in 0.01 M phosphate buffered saline (PBS) for 30 min before use. A solution of protein (1 µM, final concentration) was mixed with the drug solution (2 µM in 5% DMSO/PBS, final concentration) or vehicle. After 1 h incubation at room temperature, 90 µL of the mixture was immobilized on the membrane using a 96-well Bio-Dot Microfiltration Apparatus under vacuum. Each strip including 5–6 duplications was assigned to the corresponding antibody recognition for the epitope of interest (EOI). Briefly, the membrane was blocked with 5% nonfat milk for 1 h at room temperature and incubated with a primary antibody (1 : 2000 diluted with 5% nonfat milk) at 4 °C overnight. After washing with 0.1% Tween 20 in TBS (TBS-T) for 3 × 10 min, the membrane was incubated with HRP-conjugated secondary antibody (1 : 2000 diluted with 5% nonfat milk) for 1.5 h at room temperature and washed with TBS-T for 3 × 10 min. Visualization of EOI was performed with an enhanced chemiluminescent reagent (Pierce ECL Western Blotting Substrate) using an IVIS®Spectrum imaging system (PerkinElmer, Hopkinton, MA) with a blocked excitation filter and an opened emission filter. The chemiluminescence readout from the control group (without the tested drug) was normalized to 1.0, and the alteration effect for the EOI was quantified using the ratio between the two groups.

### Dot blot assay with cell media and brain lysates

See the ESI.†

### Zeta potential test

Aβ_1–40_ aggregates (400 nM, final concentration) in 1 mL double distilled water were mixed with the compound solution (800 nM, final concentration in distilled water) and incubated for 1 h at room temperature. The mixture was then pipetted into a folded capillary zeta cell (Malvern Panalytical) and the zeta potential was measured by using a Nano-sizer (Malvern, Nano-ZS).^20^

### ANS fluorescence assay

Aβ_1–40_ monomers (5 µM, final concentration) were incubated with or without the compound solution (10 µM, final concentration) for 1 h and the fluorescence intensity was measured by using an F-7100 fluorescence spectrometer (Hitachi, Japan). To the samples, 1-anilinonaphthalene-8-sulfonic acid (ANS) was then added, incubated for 3 min, and subjected to the same fluorescence assay procedures (*n* = 3). The final fluorescence intensities of the samples were normalized by deducting the intensity of the original samples without ANS treatment. The fluorescence intensity *versus* ANS concentration was plotted. The slopes of the two lines and the *P* value (two-tailed) were calculated using GraphPad Prism 8.0 as the parameter to compare the hydrophobicity between the two groups.^27^*P* value less than 0.05 is considered as significantly different.

### Docking studies

The structures of the Aβ_1–40_ fibrils were extracted from the RCSB Protein Data Bank (PDB: 5OQV and 2LMO), followed by the refining of the molecular properties using the Dock Prep function found in UCSF Chimera (UCSF Chimera, version 1.13.1) which included the addition of hydrogen atoms, deletion of the solvent, and assigning of the AMBER ff14SB force field for standard residues. Structures of the compounds were generated on an IQmol (IQmol, version 2.11), and minimized with the UFF force field. The docking was performed using AutoDock Vina 1.1.2 visualized on UCSF Chimera with the default parameters. The receptor search volume was set to contain the entire Aβ fibril based on their surface structure on UCSF Chimera. Each ligand docking returned the top 9 binding poses ordered based on the score function and the best docking pose and scoring values were extracted. Each docking was repeated in triplicate to assure results. The hydrophobic surface was depicted using UCSF Chimera based on the Kyte–Doolittle scale with blue being the most hydrophilic, and red being the most hydrophobic.^61^

### Molecular dynamics simulations

MD simulations of the Aβ_1–40_ monomer bound to CRANAD-17 (denoted as Aβ_1–40_-CRANAD17) in water was performed. Five independent simulations with different initial configurations and velocities were conducted using the GROningen MAchine for Chemical Simulations (GROMACS), version 2018.4, package to improve statistical efficiency.^34^ The force field parameter of CRANAD-17 was generated using the Automated forced field Topology Builder (ATB) version 3.0 web server as it provided a good estimation for drug-like novel molecules based on the GROMOS 54a7 force field. SPC water model was used as the default water model for GROMOS 54a7. Aβ_1–40_ monomer was built from a fully extended structure with amine and carboxyl groups placed at N and C termini, respectively. The protonation state of the peptide was assessed using an H++ web server at pH = 7.4, consistent with experimental conditions. The extended Aβ_1–40_ was first solvated in a cubic box filled with more than 147 000 SPC water molecules, and NaCl was added to match 0.15 M concentration to mimic the experimental conditions. Moreover, 3 additional Na^+^ ions were added to neutralize the charges. The solvated system went through the 5000-step steepest descent (SD) energy minimization. The energy minimized system underwent a 500 ps MD simulation at high temperature (700 K) under the NVT ensemble with the Berendsen thermostat (0.1 ps coupling constant) to collapse each extended system; a similar protocol was applied by Rosenman *et al.* for obtaining the collapsed Aβ_1–40_ configuration.^62^ All five collapsed configurates of Aβ_1–40_ peptides were obtained using the same protocol. From the resulting collapsed configuration, a new simulation box was constructed with about 13 000 SPC water molecules and 0.15 M NaCl concentration with 3 additional Na^+^ to balance the charges. For each newly constructed system, a CRANAD-17 molecule was randomly inserted. All systems first underwent 5000 steps SD energy minimization followed by two equilibration processes. The first equilibration was performed under the NVT ensemble for 500 ps using a Berendsen thermostat with a coupling constant of 0.1 ps to maintain the temperature at 300 K, and additional position restraints were added on all heavy protein atoms. The second equilibration was preformed using the NPT ensemble with all restraints removed for 20 ns using the v-rescale thermostat with a coupling constant of 0.5 ps and the Berendsen barostat with a coupling constant of 1.0 ps to maintain the temperature and pressure at 300 K and 1 bar respectively. The production simulation was then conducted under the NPT ensemble with hydrogen atoms being constrained using LINCS for 500 ns using the same thermostat and barostat as those in the second equilibration process. All simulations were subjected to periodic boundary conditions with an integration time step of 2 fs. The neighbor list was cut off at 1.2 nm using the Verlet scheme. Long-range electrostatic interactions were calculated with the smooth particle mesh Ewald method with a cutoff of 1.2 nm, and non-bonded van der Waals interactions were computed using a plain cutoff distance of 1.2 nm.

### Förster resonance energy transfer (FRET) studies

10 µL of PE labelled goat-anti-mouse secondary antibody (1 : 500 diluted with PBS) were incubated with primary antibody (4G8) with or without 10 µL Aβ_1–40_ monomers (final concentration: 2.5 µM) for 1 h. The mixtures were titrated with different concentrations of CRANAD-3 (0∼3 µM) and fluorescence spectra were measured by using an F-7100 fluorescence spectrometer (Hitachi, Japan). The final spectra were normalized with the control group, that is, the group without adding Aβ_1–40_ monomers.

### SPEED for small library screening

A collection of approved drugs was purchased from TargetMol and 30 synthetic compounds were provided by Dr Changning Wang's lab. A library of 1047 compounds (2 µM in 5% DMSO/PBS, final concentration) were mixed with Aβ_1–40_ monomer solution (1 µM, final concentration). After 1 h incubation at room temperature, the mixture was subjected to routine dot blot assays with LVFFAEDV as the EOI (*n* = 2 in each assay, assay performed at least in duplicate). The compounds with average luminescence intensity beyond 3SD were selected as hits.

### *In vitro* solution test with obatoclax

Aβ species were prepared according to the previously reported protocol. The fluorescence spectrum of obatoclax (250 nM) with or without Aβ species (250 nM) was recorded with excitation at 520 nM and emission from 540 nm to 900 nm by using an F-4500 fluorescence spectrophotometer (Hitachi). For black plate studies, the fluorescence spectrum of obatoclax (250 nM) with or without Aβ species (250 nM) was recorded with an IVIS®Spectrum imaging system (Caliper LifeSciences, PerkinElmer, Hopkinton, MA), and data analysis was conducted using LivingImage® 4.2.1 software.

### *In vitro* histological staining

Brain slice from a 14 month-old APP/PS1 mouse was rinsed in PBS and then incubated with 1 µM obatoclax solution (20% ethanol) for 20 minutes. The slice was washed twice for 5 minutes in 50% ethanol and twice for 5 minutes in distilled water. Next, the slice was co-stained with 0.1% thioflavin S (50% ethanol), and then washed with 80% ethanol for 1 min and 70% ethanol for 1 min. After washing with distilled water, the slices were mounted with fluorescent mounting medium and stored in a dark box at 4 °C until observation and analysis. Fluorescence images were observed using a Nikon Eclipse 50i microscope.

### *Ex vivo* histological observation

9 month-old 5xFAD mice and wild-type mice were intravenously injected with 100 µL obatoclax solution (1 mg mL^−1^, 5% DMSO, 5% cremorpho1, 90% PBS) *via* the tail vein and sacrificed at 60 minutes post-injection. The brains were harvested and cut into 10 µm slices and then co-stained with 0.1% thioflavin S (50% ethanol). After washing the slices with 80% ethanol for 1 min, 70% ethanol for 1 min and distilled water for 2 × 1 min, the fluorescence images were captured by using a Nikon Eclipse 50i microscope.

### *In vivo* NIRFOI study

*In vivo* NIR imaging was performed using an IVIS®Spectrum animal imaging system (PerkinElmer, Hopkinton, MA). 9 month-old mice (female transgenic 5xFAD, *n* = 3 and age-matched female wild type control mice, *n* = 4) were injected intravenously with an obatoclax solution (100 µL, 1 mg mL^−1^, 5% DMSO, 5% cremorphol, 90% PBS) *via* the tail vein. Sequence images were captured at 0, 5, 10, 15, 30 and 60 min after injection following the parameters: *E*_x_/*E*_m_ pairs: 535/580 nm, 535/600 nm, 570/620 nm and 570/640 nm, exposure time is auto, FOV = C. Spectral unmixing was performed with Living Image 4.2.1 software, and the Library Unmixing Method was selected.

### Statistical analyses

Quantitative data shown as mean ± s.e.m. were analyzed and presented with GraphPad Prism 8.0 software. *P* values were determined by unpaired two-tailed Student's *t*-tests to evaluate the difference between the two groups. The differences were considered significant when *P* ≤ 0.05.

## Author contributions

C. R. and B. Z. designed the project. B. Z. developed and validated the screening platform. B. Z., C. Z, Y. L. and K. Y. contributed to cell studies. B. Z., J. Y., R. T., X. S., S. H. C, C. Z., and K. R. contributed to animal studies. R. V., Y. S., Y. Y., and L. S. contributed to molecular docking and molecular dynamics studies. W. Y. synthesized and characterized the compounds. B. Z., F. Y., C. W., and A. Y. contributed to the small molecule library screening. Y. L. contributed to protein preparation. B. Z. contributed to automated dot blot, MSD, and Simoa assays. C. R. and B. Z. analyzed the results, prepared the manuscript, figures and ESI.† C. R., C. Z, C. Y. and Z. Z. provided reagents, materials, and analysis tools, and supervised the work. All authors contributed to the discussion and editing of the manuscript.

## Conflicts of interest

The authors declare no competing financial interest.

## Supplementary Material

SC-013-D2SC02819K-s001
