# Supplementary material for: Epitope alteration by small molecules and applications in drug discovery
Source: Chem Sci. 2022 Jun 28;13(27):8104–16. doi: 10.1039/d2sc02819k (PMC9278120; doi:10.1039/d2sc02819k)
Supplement: SC-013-D2SC02819K-s001 [file SC-013-D2SC02819K-s001.pdf]

Electronic Supplementary Material (ESI) for Chemical Science.
This journal is © The Royal Society of Chemistry 2022

Supporting Information

Epitope alteration by small molecules and applications in drug discovery

Biyue Zhu[1,2], Jing Yang[1], Richard Van[3], Fan Yang,[1] Yue Yu,[4] Astra Yu[1], Kathleen Ran[1], Keyi Yin[1], Yingxia Liang[5], Xunuo Shen[5], Wei Yin[1], Se Hoon Choi[5], Ying Lu[6], Changning Wang[1], Yihan Shao[3], Rudolph E. Tanzi[5], Liang Shi,[4] Can Zhang[5]*, Yan Cheng[2]*, Zhirong Zhang[2], and Chongzhao Ran[1]*

[1]Athinoula A. Martinos Center for Biomedical Imaging, Department of Radiology, Massachusetts General Hospital/Harvard Medical School, Charlestown, Boston, Massachusetts, USA, 02129; [2]Key Laboratory of Drug Targeting and Drug Delivery Systems, West China School of Pharmacy, Sichuan University, Chengdu, 610041, China; [3]Department of Chemistry and Biochemistry, University of Oklahoma, Norman, OK, 73019; [4]Department of Chemistry and Chemical Biology, University of California, Merced, California 95343, United States; [5]Genetics and Aging Research Unit, McCance Center for Brain Health, MassGeneral Institute for Neurodegenerative Disease, Department of Neurology, Massachusetts General Hospital/Harvard Medical School, Charlestown, Boston, Massachusetts, USA, 02129; [6]Department of Systems Biology, Harvard Medical School, Boston, Massachusetts, USA, 02115.

Table of Contents

Experimental Section. **4**

Supplementary Figure 1. Epitope alteration effect of crown ethers **8**

Supplementary Figure 2. Epitope alteration effect of WL12 for PD-L1 .... **10**

Supplementary Figure 3. Epitope alteration effect of CRANAD-17 for different species of Aβs **11**

Supplementary Figure 4. Negligible epitope alteration effect of ThT for Aβs detected by 4G8 and 6E10 antibodies **12**

Supplementary Figure 5. Concentration dependent profile of CRANAD-17 .... **13**

Supplementary Figure 6. Hydrophobicity change of Aβs upon interacting with CRANAD-17 .... **14**

Supplementary Figure 7. Epitope alteration effect of CRANAD-17 in biologically relevant environment **15**

Supplementary Figure 8. Epitope alteration effect of CRANAD -25, -44, -102 for Aβs **16**

Supplementary Figure 9. Docking studies of CRANAD-17 and Thioflavin T (ThT) **17**

Supplementary Figure 10. Mechanism Studies for the epitope enhancing effect of CRANAD-17 .... **18**

Supplementary Figure 11. Dot blot assay for identifying epitope enhancers for tau proteins **19**

Supplementary Figure 12. Chemical structures of positive hits in the SPEED test.. **20**

Supplementary Figure 13. The binding of GNF-5837 to Aβs 21

Supplementary Figure 14. Competitive binding of GNF-5837 and CRANAD-3 to Aβ species 22

Supplementary Figure 15. Epitope alteration effect of obatoclax for different species of Aβs 23

Supplementary Figure 16. Docking studies of obatoclax 24

Supplementary Figure 17. SPEED with Z' factor test, MSD and Simoa technology 25

Supplementary Figure 18. Epitope alteration effect with auto-antibody 26

Supplementary Figure 19. Epitope alteration effect of CRANAD-17 for different species of Aβs by using 12F4 antibody 27

Supplementary Table 1. Autodock Vina Score of Compounds upon Interacting with Aβ 28

Supplementary Table 2. Samples information of Human Serum................................ 29

References... 30

Experimental Section.

Fluorescence polarization (FP) assays. A solution of Alexa fluor 488 labeled primary or secondary antibody (1:2000 diluted with PBS buffer) was mixed with compound solution (2 μM in 5% DMSO/PBS, final concentration) or vehicle and incubated for 1 h at room temperature. The mixture was transferred to a black 96 well plates and the FP signal in millipolarization (mP) units was measured by a SpectraMax microplate reader (Molecular Devices). The raw mP signal from the control group (without tested compound) was normalized to 1.0, and signal change was quantified using the ratio between two groups.

7PA2 Cell Culture. The Chinese hamster ovary CHO cell line stably expressing *Indiana* mutation in APP, also known as 7PA2 cells, was cultured in Dulbecco's Modified Eagle's Medium/F-12 medium with high glucose (Gibco, USA) supplemented with 2 mM L-glutamine, 200 μg/ml G418, 100 IU/mL penicillin, and 100 μg/mL streptomycin at 37 °C in an atmosphere containing 5% CO_2.[1-3]

Aβs Concentration Measurement in Cells and Mice brain. 7PA2 cells were seeded at 4 × 10^5 in a 6-well cell culture plate (Costar). After 24 h incubation, the cell media was collected and Aβs ($Aβ_{1-40}$, $Aβ_{1-38}$ and $Aβ_{1-42}$) concentration were quantified using the MSD triplex assay via an electrochemiluminescence-based multi-array method.[4, 5] The brain of APP/PS1 transgenic mouse was homogenated with RIPA lysis buffer and incubated on ice for 30 min, followed by centrifuging at 14000 rpm for 15 min at 4 °C. The Aβs ($Aβ_{1-40}$, $Aβ_{1-38}$ and $Aβ_{1-42}$) concentration in supernatants were analyzed with MSD triplex assay by following the manufacturer protocols.

Dot Blot Assay with Cell Media and Brain Lysates. Nitrocellulose membrane (0.2 μm, Bio-Rad) was soaked in 0.01 M phosphate buffered saline (PBS) for 30 min before use. The cell media of 7PA2 cells and brain homogenate of APP/PS1 transgenic mouse with or without CRANAD-17 (1-50 nM in 10% DMSO/PBS, final concentration) were immobilized on the nitrocellulose membrane and subjected to routine dot blot test.

Fluorescence Spectra Measurement. 250 nM of Aβ species were added to CRANAD-3 solution (final concentration, 250 nM in 1% DMSO/PBS) . The fluorescence spectra were scanned by a F-7100 fluorescence spectrometer (Hitachi, Japan) with excitation at 560 nm and emission from 590-900 nm. Next, a solution of GNF-5837 was spiked to the mixture and scanned at 5 min and 15 min after compound addition. The fluorescence emission spectra of CRANAD-3 without Aβs was also scanned under the same parameter.

Dot Blot Assay with Auto-antibodies. Healthy human serum and AD patient serum were commercial available samples purchased from Innovative Research. The patients were clinically identified by Innovative Research and detailed information was documented in the Supplementary table 2. Four individuals were included for each cohort. Serum from health individuals or AD patients were diluted in 5% nonfat milk (1: 150) as primary antibody solution. Dot blot assays were conducted by following routine protocols.

Z' factor Assays. Nitrocellulose membrane (0.2 μm, Bio-Rad) was soaked in 0.01 M phosphate buffered saline (PBS) for 30 min before use. A solution of protein (1 μM, final concentration) was mixed with drug solution (4 μM in 5% DMSO/PBS, final

concentration) or vehicle. After 1 h incubation at room temperature, 90 μL of mixture was immobilized on the membrane using a 96-well Bio-Dot Microfiltration Apparatus under vacuum. The plate was divided into half negative control and half positive control (GNF-5837) and subjected to routine dot blotting by using 4G8 antibody for detection. The Z' factor was calculated by the following equation:

$$Z' = 1 - \left| \frac{3\sigma_{+} + 3\sigma_{-}}{\mu_{+} - \mu_{-}} \right|$$

The parameters are the means (μ) and standard deviations (σ) of both positive (+) and negative (-) controls.

Meso Scale Discovery (MSD) Assays. Aβ Peptide Panel 1 (4G8) Kit was used for detecting $A\beta_{1\text{-}40}$ in a 96 well MSD plate. Briefly, 150 μL of diluent 35 was added to each well of 96 well MSD plate and incubated for 1 h at room temperature. A solution of $A\beta_{1\text{-}40}$ monomers was mixed with compound solution (Aβ = 200 pg/ml, compound = 100 pM final concentration) or vehicle and incubated for 1 h at room temperature. The plate was washed with MSD buffer for 3 times followed by adding 25 μL of detection antibody solution and 25 μL of sample solution or calibrators to each well of MSD plate. After 2 h incubation with shaking, the plate was washed with MSD buffer for 3 times followed by adding 150 μL of 2X reading buffer to each well and analyzing the plate by a SECTOR® MSD instrument (USA).

Single Molecule Array (Simoa). Simoa assays were conducted at Quanterix demo lab. Briefly, a solution of $A\beta_{1\text{-}40}$ monomers was mixed with compound solution (Aβ = 1 pg/ml, compound = 0.5, 2.5 pM, final concentration) or vehicle and incubated for 1 h at room temperature. 25 μL capture antibody-coated paramagnetic beads were added to

a 96 well plate, followed by adding 100 μL samples, calibrators or controls and 20 μL detection antibody. After incubating for 1 h at room temperature with a Simoa microplate shaker, the plate was washed with a Simoa microplate washer for 3 times. Next, 100 μL streptavidin-β-galactosidase reagent was added to each well and incubated for 10 min with a Simoa microplate shaker. After washing the plate a Simoa microplate washer, the beads were resuspended in a resorufin β-D-galactopyranoside substrate solution and transferred to the Simoa Disc. The average enzymes per bead (AEB) was determined digitally with time-lapsed fluorescence intensity quantification.

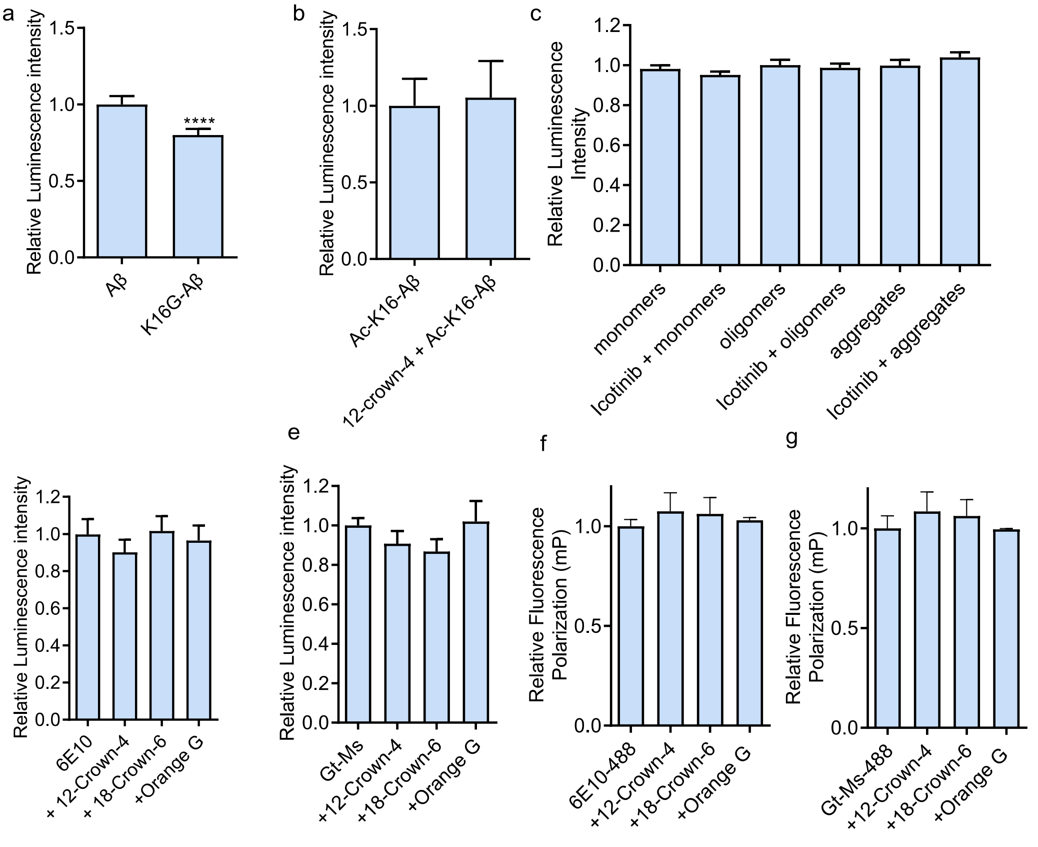

Supplementary Figure 1 | Epitope alteration effect of crown ethers. a) Quantitative analysis of dot blot assay for 1 μM native Aβs and mutated Aβs (K16G-Aβ) detected by 6E10 antibody (n = 6). Mutated Aβs showed decreased signals compared with non-modified Aβs, indicating the positive charge on K16 is critical for 6E10 antibody recognition. **b**) Quantitative analysis of dot blot assays for 1 μM acetylated-K16 Aβs (Ac-K16-Aβ) with or without 12-crown-4 (2 μM) by using 6E10 antibody (n = 6). The addition of 12-crown-4 showed negligible change of readout of Ac-K16-Aβ, further suggesting the interaction of 12-crown-4 and K16. **c**) Relative luminescence intensity of dot blot assays for 1 μM $A\beta_{1-40}$ monomers, oligomers and aggregates with or without 2 μM Icotinib by using 6E10 antibody (n ≥ 6 for each group). Icotinib did not show significant alteration effect. **d,e**) Relative luminescence intensity of HRP labeled 6E10

antibody (**d**) and secondary antibody (Gt-Ms) (**e**) before and after adding compounds. No significant change was found, indicating the alteration effects of crown ethers in dot blot assays are due to interaction with target proteins and not antibodies. **f,g)** Fluorescence polarization assay of Alexa fluo 488 labeled 6E10 (**f**) or secondary antibody (Gt-Ms-488) (**g**) before and after adding compounds. No significant readout change was found, indicating the signal changes from dot blot assays were resulted from Aβ epitope alteration.

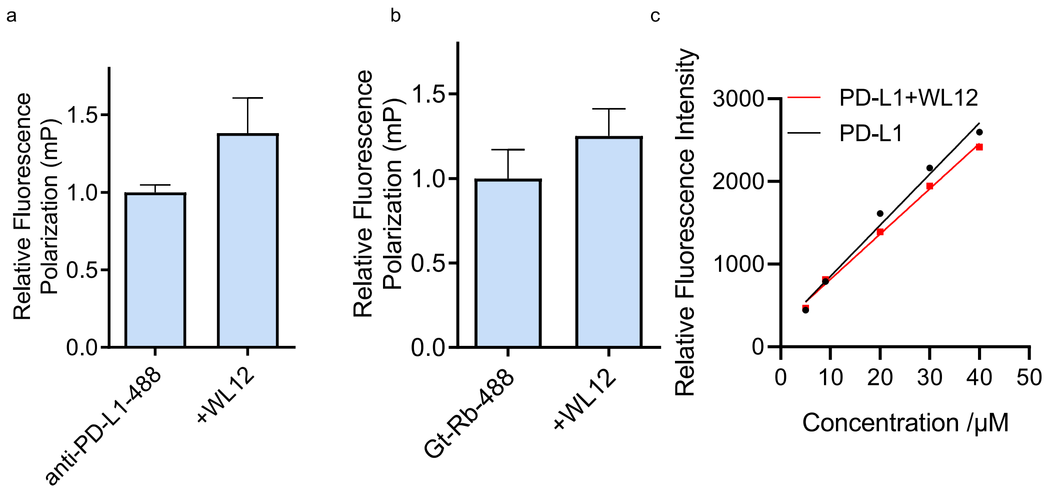

Supplementary Figure 2 | Epitope alteration effect of WL12 for PD-L1. a,b) Fluorescence polarization assay of Alexa fluo 488 labeled anti-PD-L1 antibody (**a**) or secondary antibody (Gt-Rb-488) (**b**) with or without WL12 peptide. Negligible readout change was found, indicating the signal changes from dot blot assays were resulted from epitope alteration. **c)** Normalized fluorescence intensity of hydrophobicity probe ANS (0-40 μM) after adding 5 μM PD-L1 with or without 10 μM WL12. The curve showed significantly changed slope (P=0.0056), indicating surface hydrophobicity change upon binding with WL12.

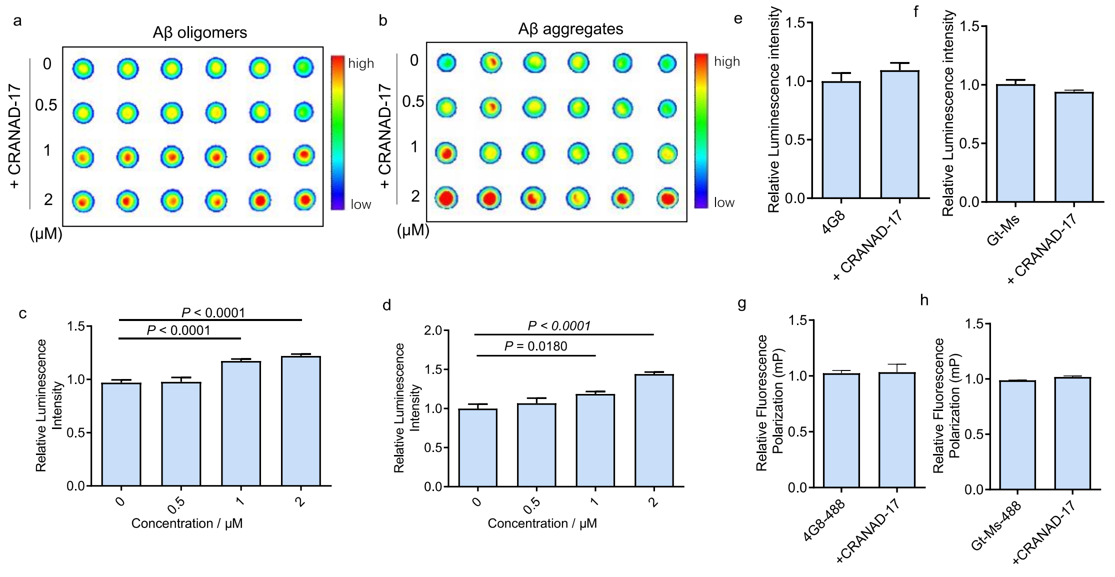

Supplementary Figure 3 | Epitope alteration effect of CRANAD-17 for different species of Aβs. a-d) Epitope alteration assays via dot blotting and quantitative data of 1 μM oligomers (**a,c**) and aggregates (**b,d**) with different concentrations of CRANAD-17 by using 4G8 antibody. The data showed significantly increased signal of all Aβ species after interacting with CRANAD-17 (The data of Aβ monomers were shown in Fig. 2**b,c**). **e,f**) Relative readout of 4G8 antibody (**e**) and secondary antibody (Gt-Ms) (**f**) before and after adding CRANAD-17. No significant change was found, indicating the alteration effects of CRANAD-17 in dot blot assays are due to interaction with target proteins and not antibodies. **g,h**) Fluorescence polarization assay of Alexa fluo 488 labeled 4G8 (**g**) or secondary antibody (Gt-Ms-488) (**h**) before and after adding CRANAD-17. No significant readout change was found, indicating the signal changes from dot blot assays were resulted from Aβ epitope alteration.

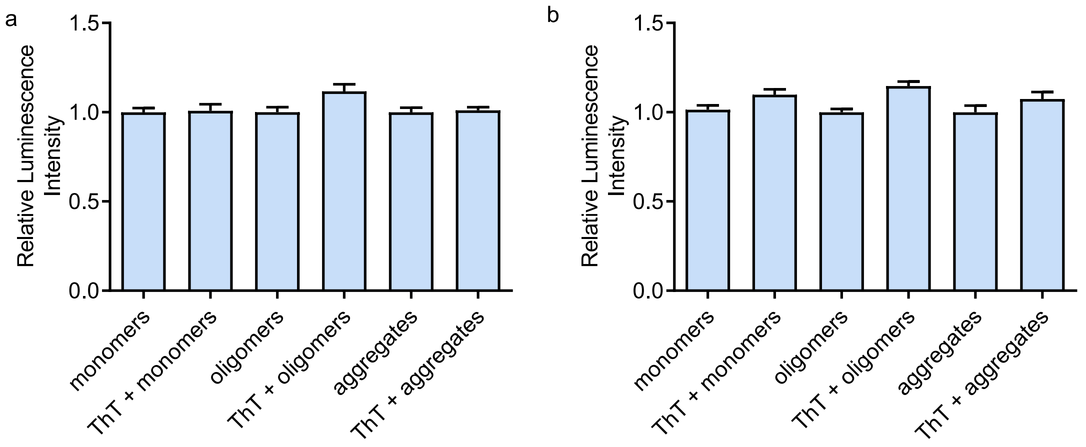

Supplementary Figure 4 | Negligible epitope alteration effect of ThT for Aβs detected by 4G8 and 6E10 antibodies. Relative luminescence intensity of epitope alteration assays for 1 μM Aβ monomers, oligomers, aggregates with or without ThT (2 μM) by using 4G8 (**a**) and 6E10 (**b**) antibody (n ≥ 6 for each group). The data showed no apparent changes of the signal from Aβ species with and without ThT.

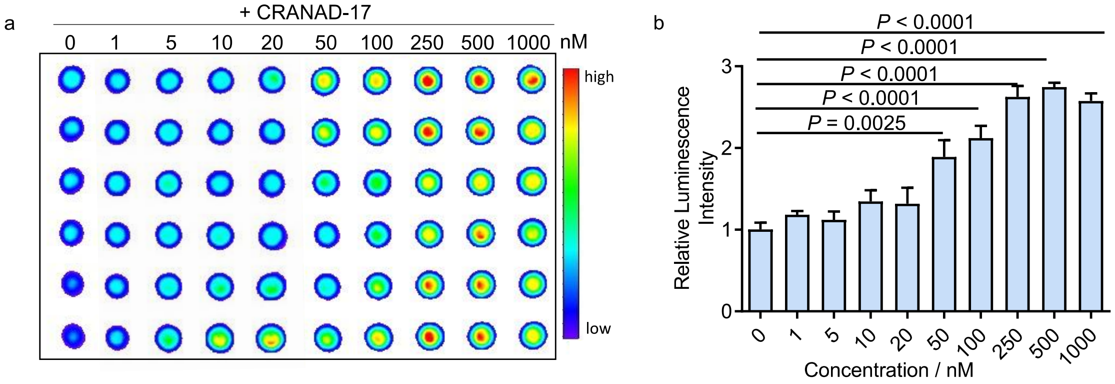

Supplementary Figure 5 | Concentration dependent profile of CRANAD-17. Epitope alteration assays via dot blotting (**a**) and quantitative data (**b**) of Aβs upon mixing with CRANAD-17 (0-1000 nM). The enhancement of readout was in a concentration-dependent manner.

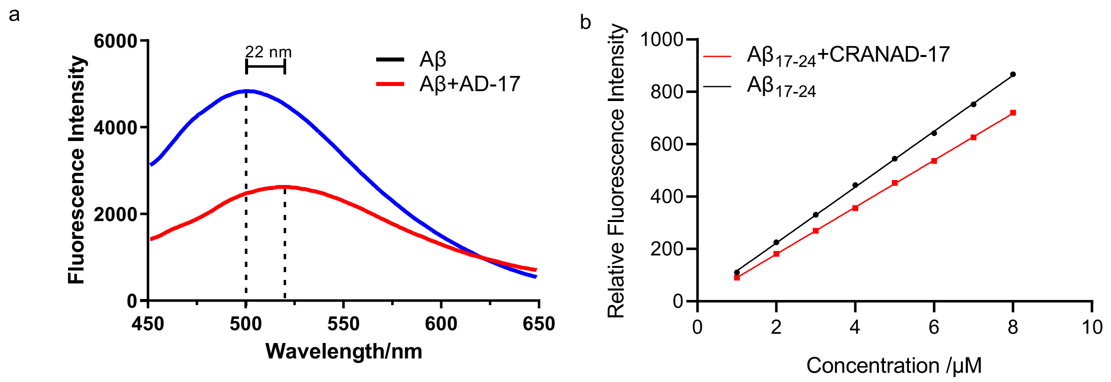

Supplementary Figure 6 | Hydrophobicity change of Aβs upon interacting with CRANAD-17. Normalized fluorescence emission spectra (**a**) and fluorescence intensity (**b**) of hydrophobicity probe ANS (1-8 μM) after adding 5 μM $A\beta_{1\text{-}40}$ (**a**) or $A\beta_{17\text{-}24}$ (**b**) monomers with or without 10 μM CRANAD-17. CRANAD-17 binding cause significant blueshift of the emission spectra of ANS when interacting with $A\beta_{1\text{-}40}$ monomer. The curve of ANS fluorescence intensity to $A\beta_{17\text{-}24}$ showed significantly changed slope (P<0.0001), indicating surface hydrophobicity change upon interacting with CRANAD-17.

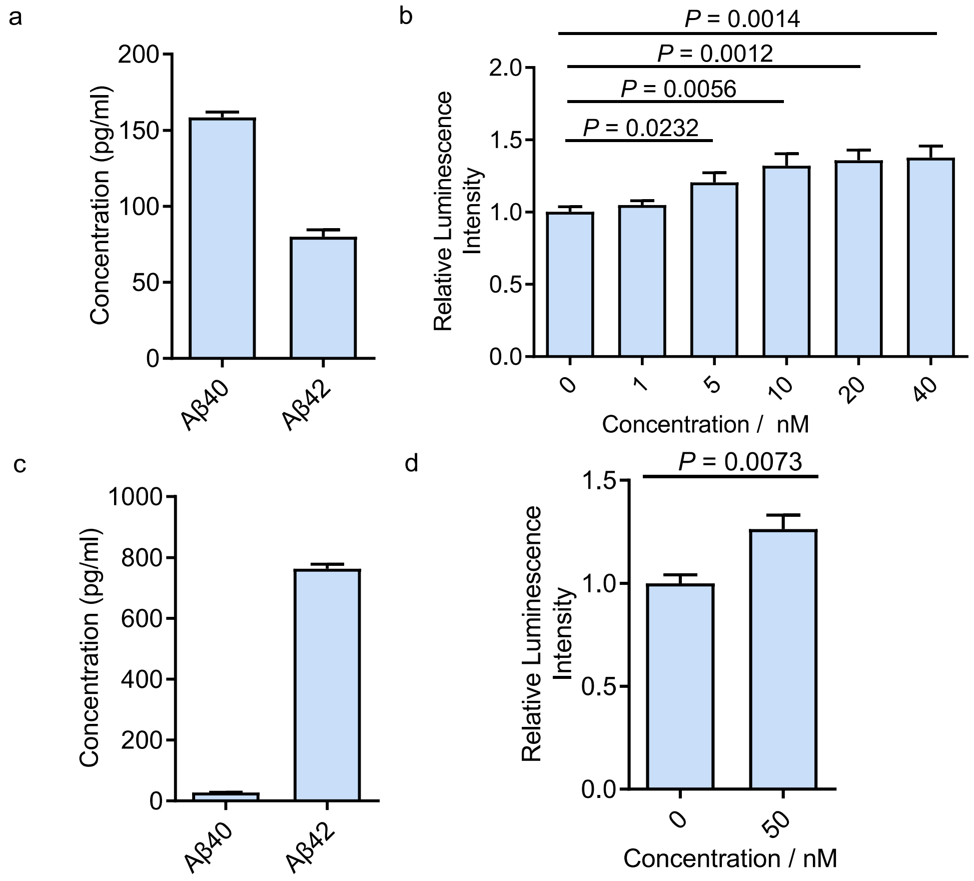

Supplementary Figure 7 | Epitope alteration effect of CRANAD-17 in biologically relevant environment. a,c) Aβ_{1-40} and Aβ_{1-42} concentration of 7PA2 cell media (**a**) and 5xFAD transgenic mice brain homogenate (**c**) were measured by meso scale discovery test by using Aβ peptide panel 1 kit. **b,d**) Quantitative data of epitope alteration assay of cell media (**b**) and transgenic mice brain homogenate (**d**) with or without CRANAD-17. The epitope alteration effect could still be detected even at picogram concentrations of Aβs.

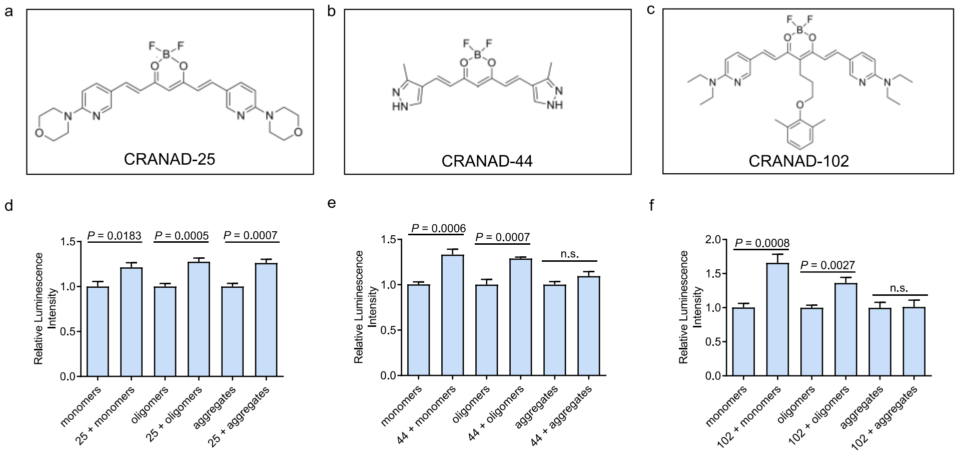

Supplementary Figure 8 | Epitope alteration effect of CRANAD -25, -44, -102 for Aβs. Chemical structure of CRANAD-25 (**a**), -44 (**b**) and -102 (**c**). Quantitative data of epitope alteration assays and for 1 μM Aβ monomers, oligomers and aggregates with (+) or without (-) 2 μM of CRANAD-25 (**d**), -44 (**e**) and -102 (**f**) by using 4G8 antibody.

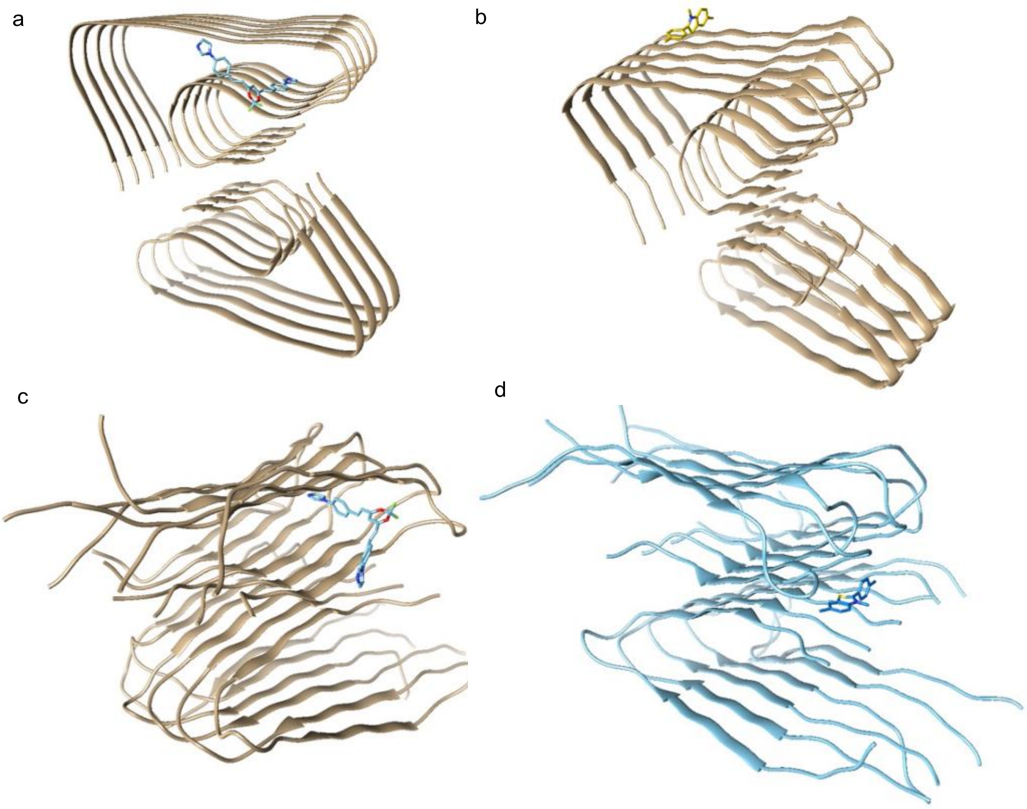

Supplementary Figure 9 | Docking studies of CRANAD-17 and Thioflavin T (ThT).

Calculated binding poses of CRANAD-17 (**a,c**) and ThT (**b,d**) for Aβ_{1-40} aggregates (PDB: 5OQV for **a-b** and PDB: 2LMO for **c-d**). CRANAD-17 displayed specific interaction within ^{17}LVFFAEDV^{24} segment in both models while ThT showed interaction at around ^{10}YEVHHQK^{16} for 5OQV model and around C-terminal in 2LMO model.

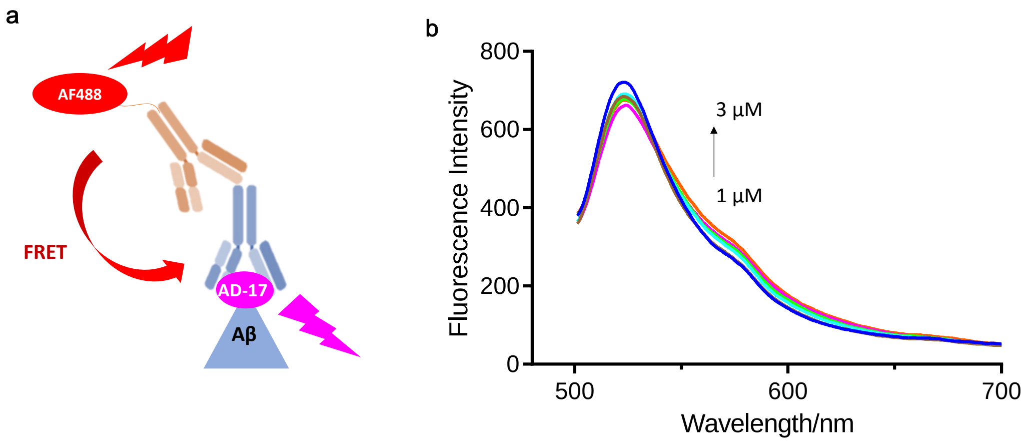

Supplementary Figure 10 | Mechanism Studies for the epitope enhancing effect of CRANAD-17. a) Illustration of FRET assay. If CRANAD-17 could bind with the epitope of 4G8 antibody on Aβ monomers and form ternary complex, the Alexa fluo488 (AF488)-labeled secondary antibody could serve as the donor molecule and excite the CRANAD-17. **b**) Fluorescence spectral of the mixture of Aβ monomers, 4G8 antibody and AF488-labeled secondary antibody titrated with CRANAD-17 (1-3 μM). FRET phenomenon was shown as the emission peaks of AF488 decreased while the emission peaks of CRANAD-17 simultaneously increased.

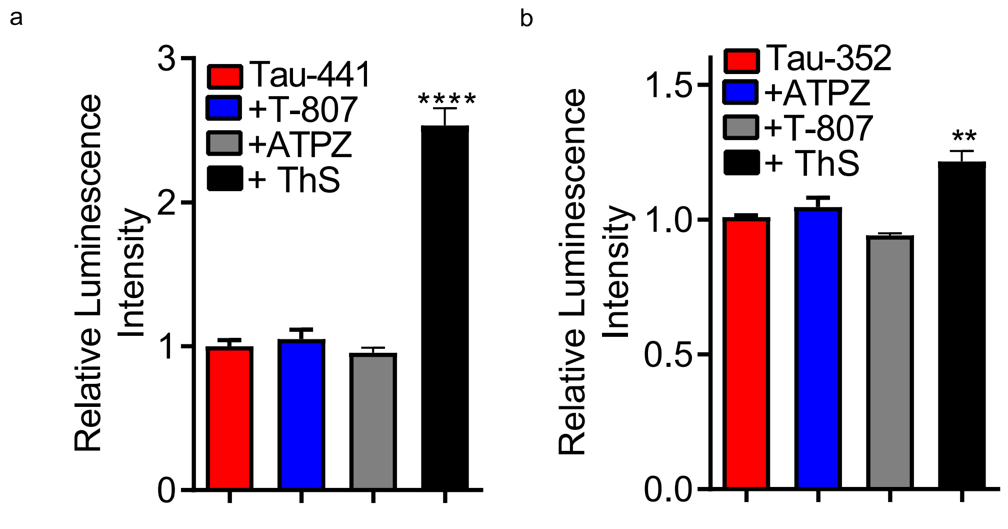

Supplementary Figure 11 | Dot blot assay for identifying epitope enhancers for tau proteins. a,b) Quantitative analysis of dot blot assay for full-length human tau protein (tau-441) (**a**) or 3-repeat tau (tau-352) (**b**) treated with T-807, ATPZ and ThS using 8E6/C11 antibody, whose epitope lies within 209-224 a.a. of human tau (3-repeat isoform RD3) containing VQIVYK segment (n = 6). T-807 and ATPZ showed negligible signal change, suggesting the interacting sites of these compounds for tau protein are not within 209-224 a.a. segment. Only ThS showed significantly increased signal, which is in accordance with previous studies that indicate ThS could bind to the VQIVYK fragment.

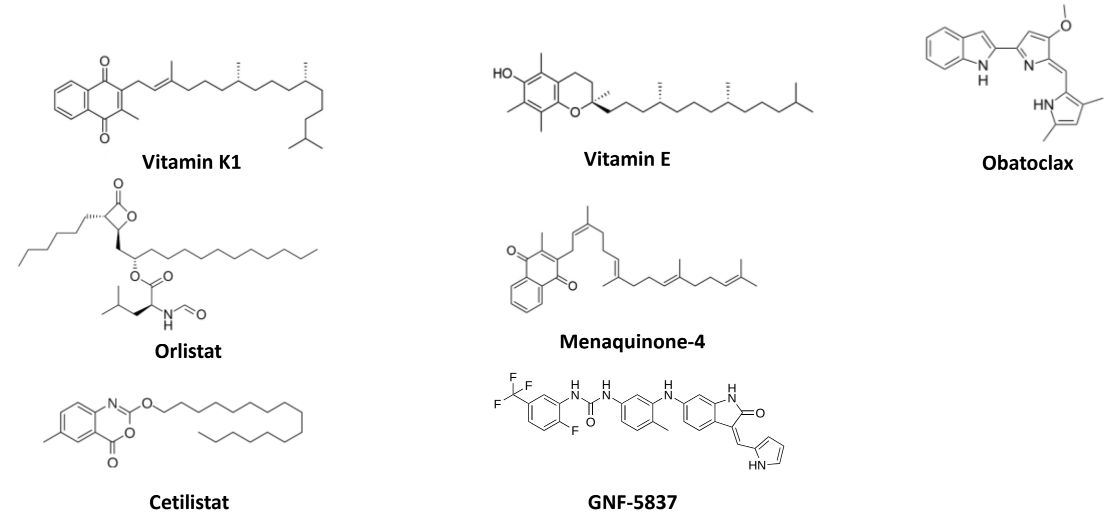

Supplementary Figure 12 | Chemical structures of positive hits in the SPEED test.

The compounds with average luminescence intensity beyond 3SD were selected as hits.

The screening results showed 7 positive results.

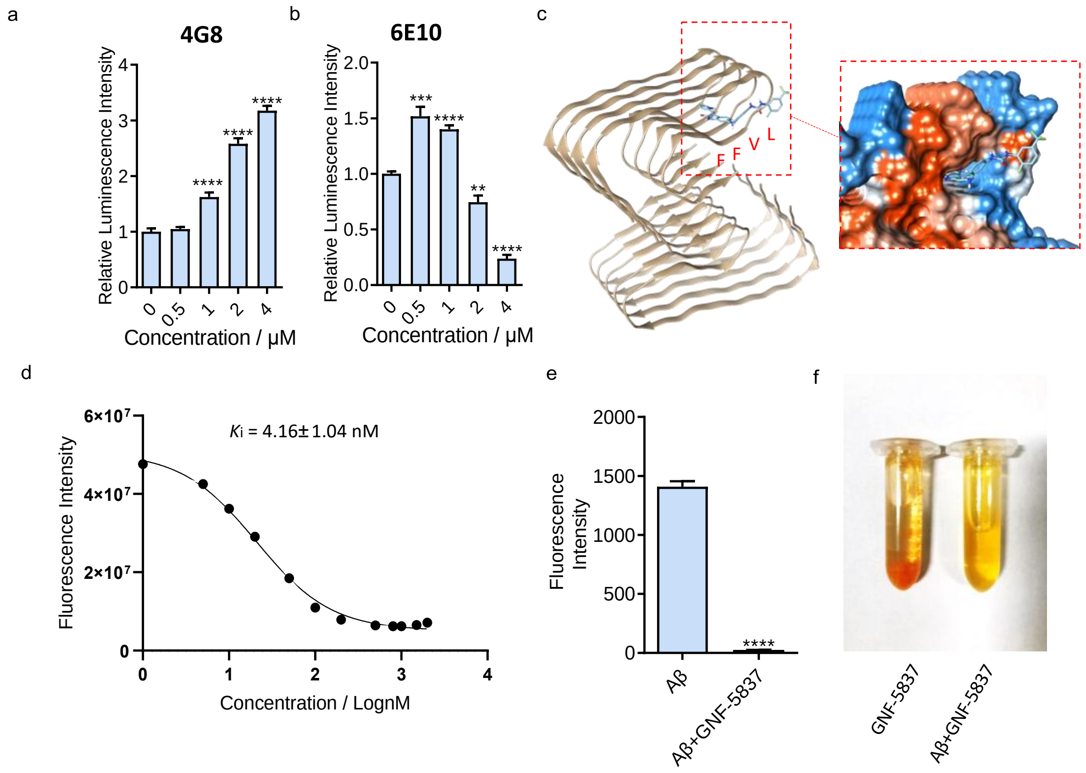

Supplementary Figure 13 | The binding of GNF-5837 to Aβs. a,b) Quantitative data of epitope alteration assay of different concentrations of GNF-5837 (0-4 μM) detected by 4G8 (**a**) or 6E10 (**b**) antibody. **c**) Docking studies of GNF-5837 upon interacting with Aβs. **d**) Competitive binding assay of Aβ monomers and CRANAD-3 with different concentrations of GNF-5837. **e**) ThT fluorescence assay for monitoring Aβ aggregation (25 μM) with or without GNF-5837 (25 μM). **f**) Image of the binding of GNF-5837 (25 μM) with or without $A\beta_{1-40}$ monomers (25 μM).

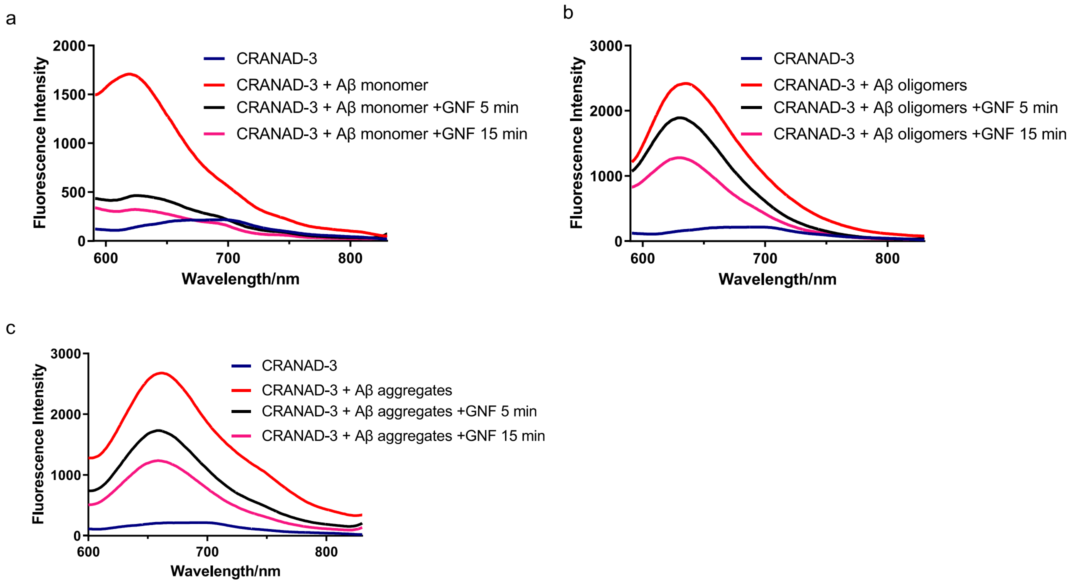

Supplementary Figure 14 | Competitive binding of GNF-5837 and CRANAD-3 to Aβ species. a-c) Fluorescence emission spectra of 250 nM CRANAD-3 and 250 nM Aβ monomers (**a**), oligomers (**b**), and aggregates (**c**) with or without 250 nM GNF-5837. CRANAD-3 showed fluorescence turn-on phenomenon when bound with Aβs and the addition of GNF-5837 significantly decreased the fluorescence intensity, indicating GNF-5837 can bind with Aβ species and inhibit the binding of CRANAD-3 to Aβs.

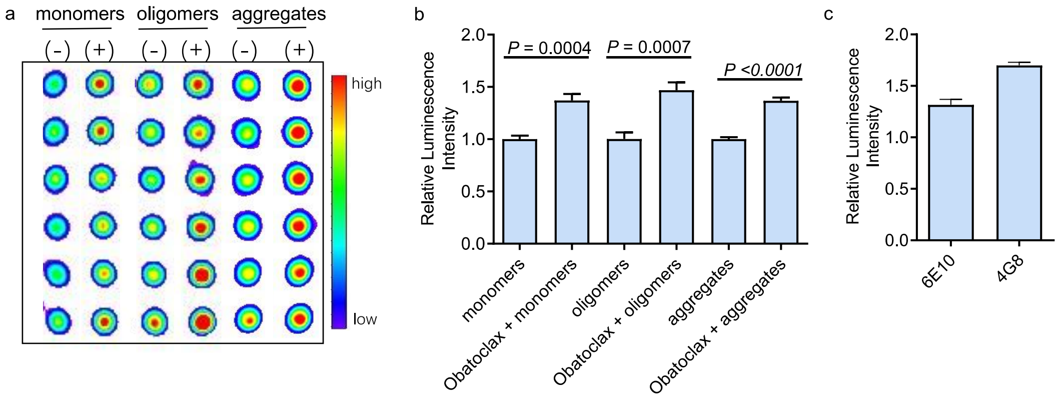

Supplementary Figure 15 | Epitope alteration effect of obatoclax for different species of Aβs. Epitope alteration assays via dot blotting (**a**) and quantitative data (**b**) of 1 μM Aβ monomers, oligomers and aggregates with (+) or without (-) 2 μM obatoclax by using 4G8 antibody. The data showed significantly increased signal from all Aβ species after interacting with obatoclax. **c**) Comparison of epitope alteration effect of 1 μM obatoclax for 1 μM Aβ monomer detected by 6E10 or 4G8 antibody.

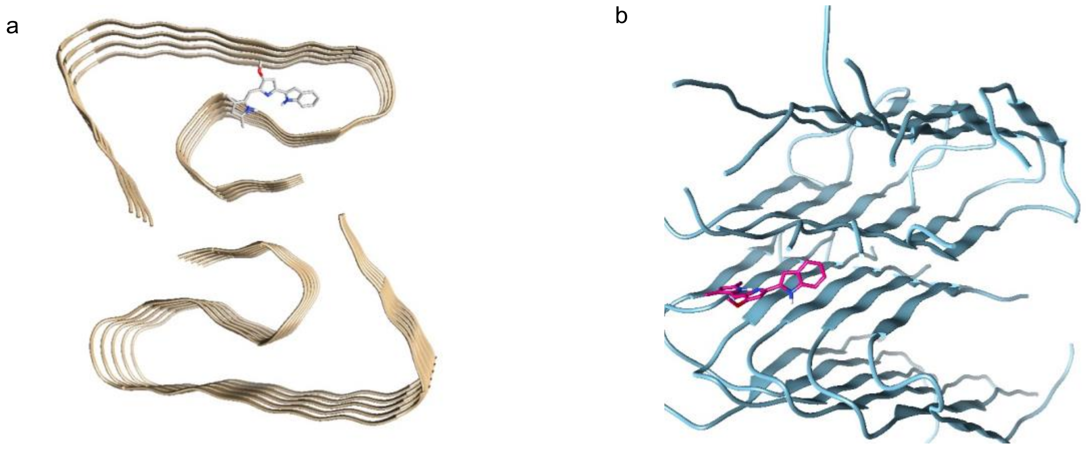

Supplementary Figure 16 | Docking studies of obatoclax. Calculated binding poses of obatoclax for A$\beta_{1\text{-}40}$ aggregates (PDB: 5OQV for **a** and PDB: 2LMO for **b**). Obatoclax showed specific interaction within ^{17}LVFFAEDV^{24} segment in 5OQV model and around C-terminal in 2LMO model.

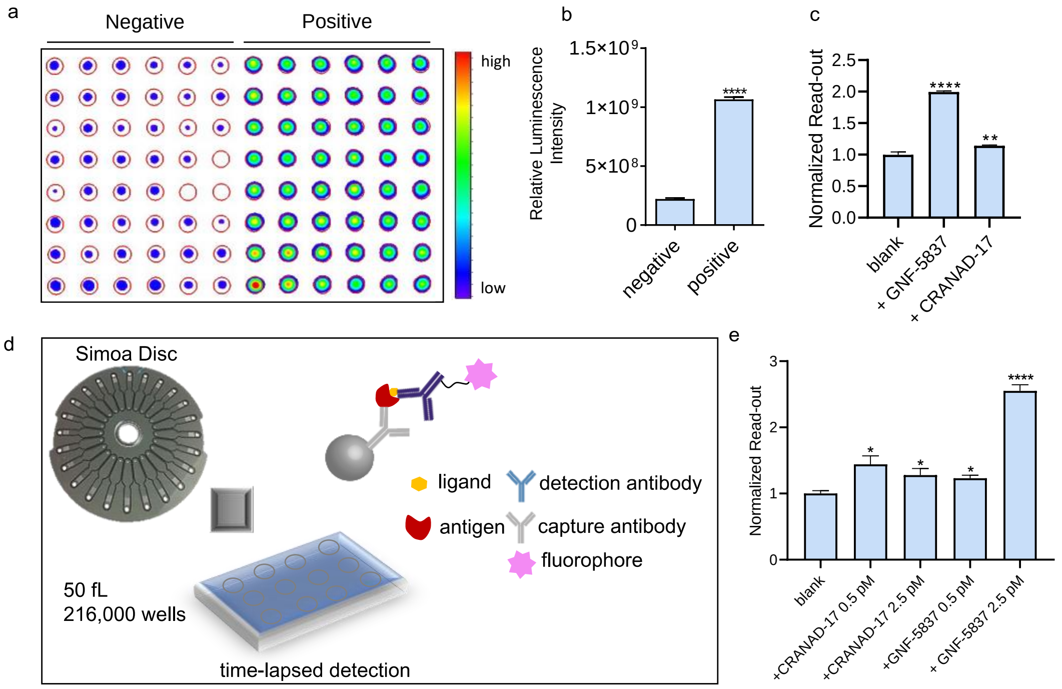

Supplementary Figure 17 | SPEED with Z' factor test, MSD and Simoa technology. **a,b**) Representative Z' factor results for Aβ_{1-40} monomers by using 4G8 antibody. Positive control group (GNF-5837) showed significantly enhanced signal compared with negative control group. **c**) Normalized readout of MSD assay for 200 pg/mL Aβ_{1-40} monomers. With addition of CRANAD-17 or GNF-5837, the antibody binding significantly enhanced. **d**) Diagram of Simoa technology. **e**) Normalized readout of Simoa assay for 1 pg/mL Aβ_{1-40} monomers with or without 0.5, 2.5 pM CRANAD-17 or GNF-5837. Both of the ligands showed obviously increased signal compared with blank control.

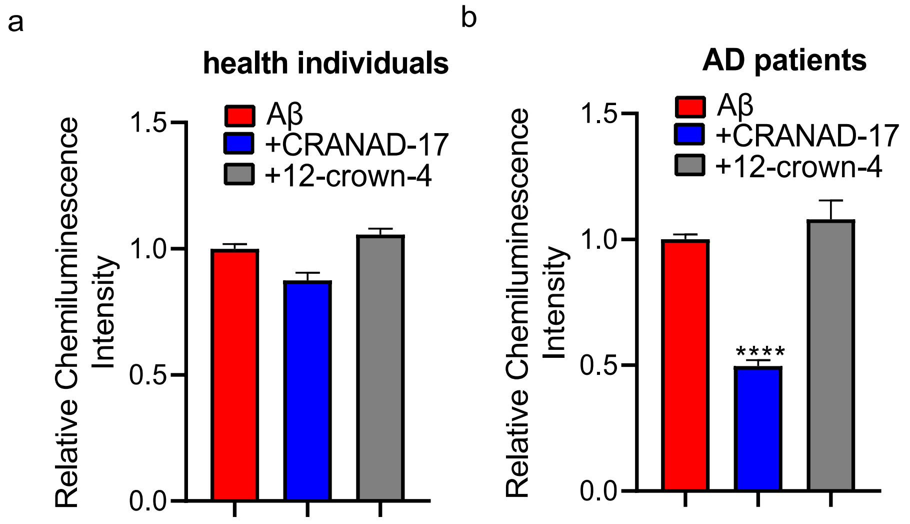

Supplementary Figure 18 | Epitope alteration effect with auto-antibody. a,b) Quantitative analysis of dot blot assay of Aβ monomers (1 μM) with 12-crown-4 (2 μM) or CRANAD-17 (2 μM) by using auto-antibodies from serum of health individuals (n = 4) and AD patients (n = 4). The results showed significantly decreased signal of AD patients group upon mixing with CRANAD-17, indicating CRANAD-17 could alter the binding between Aβs and auto-antibodies from AD patients.

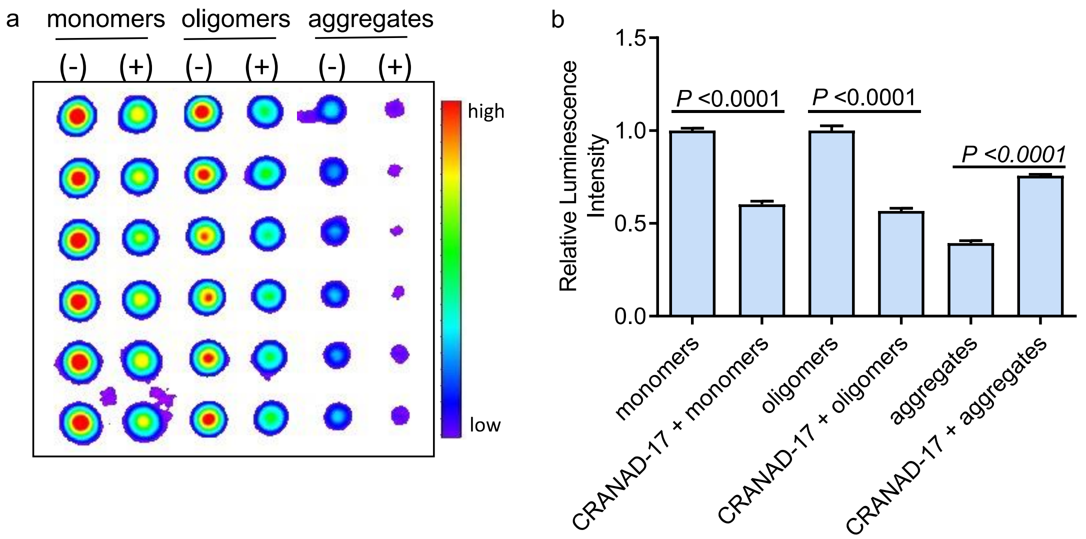

Supplementary Figure 19 | Epitope alteration effect of CRANAD-17 for different species of Aβs by using 12F4 antibody. Epitope alteration assays via dot blotting (**a**) and quantitative data (**b**) of 1 μM Aβ monomers, oligomers and aggregates with (+) or without (-) 2 μM CRANAD-17. The data showed significantly decreased signal from all Aβ species after interacting with CRANAD-17.

Supplementary Table 1 | Autodock Vina Score of Compounds upon Interacting with Aβs.

PDB code		CRANAD-17	ThT	GNF-5837	Obatoclax
5OQV	Scores[a]	-9.4	-7.2	-10.8	-7.3
	a.a. segment[b]	^{17}LVFFAEDV GSNKGAI^{31}	^{10}YEVHHQ KLV^{18}	^{17}LVFFAED VGSNKGAI^{31}	^{17}LVFFAED VGSNKGAIIG^{33}
2LMO	Scores[a]	-10.1	-7.9	-10.7	-8.6
	a.a. segment[b]	^{17}LVFFAEDV GSNKGAIIGLM^{35}	^{36}VGGVV^{40} & ^{25}GSNKGAII^{32}	binds at the interface of two aggregates	^{36}VGGVV^{40} & ^{25}GSNKGAII^{32}

[a]Docking scores (kcal/mol) were calculated by Autodock vina. [b]Calculated interacting sites within Aβ model.

Supplementary Table 2 | Sample Information of Human Serum.

Sex	Age	Ethnicity	Diagnosis
Male	88	Caucasian	Alzheimer's disease
Female	67	Hispanic	Alzheimer's disease
Male	65	African American	Alzheimer's disease
Female	68	Hispanic	Alzheimer's disease
Female	65	Hispanic	Healthy
Female	59	Black	Healthy
Male	53	Black	Healthy
Male	43	Black	Healthy

References

1. R. Dodel, K. Balakrishnan, K. Keyvani, O. Deuster, F. Neff, L. C. Andrei-Selmer, S. Roskam, C. Stuer, Y. Al-Abed, C. Noelker, M. Balzer-Geldsetzer, W. Oertel, Y. Du and M. Bacher, *J. Neurosci.*, 2011, **31**, 5847-5854.
2. A. Caccamo, S. Majumder, A. Richardson, R. Strong and S. Oddo, *J. Biol. Chem.*, 2010, **285**, 13107-13120.
3. G. Joshi, K. A. Gan, D. A. Johnson and J. A. Johnson, *Neurobiol. Aging*, 2015, **36**, 664-679.
4. E. C. Breen, S. M. Reynolds, C. Cox, L. P. Jacobson, L. Magpantay, C. B. Mulder, O. Dibben, J. B. Margolick, J. H. Bream, E. Sambrano, O. Martinez-Maza, E. Sinclair, P. Borrow, A. L. Landay, C. R. Rinaldo and P. J. Norris, *Clin. Vaccine Immunol.*, 2011, **18**, 1229-1242.
5. M. Z. Kounnas, A. M. Danks, S. Cheng, C. Tyree, E. Ackerman, X. Zhang, K. Ahn, P. Nguyen, D. Comer, L. Mao, C. Yu, D. Pleynet, P. J. Digregorio, G. Velicelebi, K. A. Stauderman, W. T. Comer, W. C. Mobley, Y. M. Li, S. S. Sisodia, R. E. Tanzi and S. L. Wagner, *Neuron*, 2010, **67**, 769-780.
